# What do patients and family-caregivers value from hospice care? A systematic mixed studies review

**DOI:** 10.1186/s12904-019-0401-1

**Published:** 2019-02-08

**Authors:** Nicole Marie Hughes, Jane Noyes, Lindsay Eckley, Trystan Pritchard

**Affiliations:** 1 School of Healthcare Sciences, Bangor University, Bangor, North Wales LL57 2DG UK; 2 Present address: North of England Zoological Society (Chester Zoo), Caughall Road, Chester, UK; 3 St Davids Hospice, Llandudno, North Wales, UK

**Keywords:** Palliative care, Systematic review, Patient, Carer, Family, Hospice, Value, quality of life

## Abstract

**Background:**

It is not known which attributes of care are valued the most by those who experience hospice services. Such knowledge is integral to service development as it facilitates opportunities for continuous improvement of hospice care provision. The objectives of this mixed-studies systematic review were to explore patients’ and their family carer views and experiences, to determine what they valued about adult hospice care in the UK.

**Methods:**

ASSIA, PubMed, CINAHL and PsycINFO were searched from inception, up until March 2017 to identify qualitative, quantitative, and mixed-methods studies. Four additional searching techniques supplemented the main search and grey literature was included. A three-stage mixed-method systematic review was conducted with a sequential exploratory design. Thematic synthesis was used with qualitative data, followed by a narrative summary of the quantitative data. The qualitative and quantitative syntheses were then juxtaposed within a matrix to produce an overarching synthesis.

**Results:**

Thirty-four studies highlighted that what patients and carers valued was generally context specific and stemmed from an amalgamation of hospice service components, which both individually and collectively contributed to improvements in quality of life. When the syntheses of qualitative and quantitative studies were viewed in isolation, the value placed on services remained relatively consistent, with some discrepancies evident in service availability. These were commonly associated with geographical variations, as well as differences in service models and timeframes. Through an overarching synthesis of the qualitative and quantitative evidence, however, notable variations and a more nuanced account of what people valued and why were more prominent, specifically in relation to a lack of social support for carers, disparate access to essential services, the underrepresentation of patients with a non-cancer diagnosis, and the dissatisfaction with the range of services provided.

**Conclusion:**

Review findings strengthen the existing evidence base and illuminates the underpinning elements of hospice care most valued by patients and their families. With large disparities in the availability of services, however, the underrepresentation of patients with non-malignant diseases and the limited evidence base demonstrating the adequate addressment of the social needs of carers, there continues to be considerable gaps that warrants further research.

**Electronic supplementary material:**

The online version of this article (10.1186/s12904-019-0401-1) contains supplementary material, which is available to authorized users.

## Background

Palliative care policy and practice has evolved continuously since its inception to enhance the lives of people with life-limiting illnesses and their families. In the United Kingdom (UK), service improvement has been informed by the National Health Services: End of life care strategy [1] which aims to ensure that whatever their diagnosis patients and families receive the best care possible. With the growing demand for palliative care due to the increasing complexity of chronic illnesses coupled with limited resources, hospices are under significant financial pressure to continually redesign services. For this reason, along with the temporal nature of the evidence and changes in practice over time, it is important to continuously identify patient and family preferences and what they value about palliative care received. A synthesis of evidence on what patients and family carers’ value about palliative care has not been conducted before. The current review is designed to address this evidence gap. The objectives of this mixed-studies systematic review were to explore patient and family-caregivers’ views and experiences and to determine what they valued about adult hospice care in the UK.

## Method

### Review design

A three-stage mixed-method systematic review was utilised, following a sequential exploratory design [2] whereby the synthesis of qualitative data using the Thomas and Harden [3] approach was followed by a narrative synthesis of quantitative data. Finally the two syntheses were integrated in an overarching synthesis.

### Search strategy

The search strategy was designed with an information scientist and the following databases were searched from inception to March 2017: ASSIA (Applied Social Sciences Abstracts), PubMed, CINAHL (The Cumulative Index to Nursing and Allied Health Literature) and PsycINFO. It was based on key words and terms from the intervention, perspective and evaluation of the SPICE framework and different techniques, such as medical subject headings (MeSh) ‘hospice’ and ‘palliative’, in conjunction with Boolean operators and truncated words which were adapted to suit the needs of each individual database searched (See Additional file 1). To aid the rigour of the search strategy, additional studies were found via four additional searching techniques. Grey literature was sought using the following relevant subject related websites; Hospice UK, NICE evidence, British Library e-theses Online Service (EtHOS) and The International Observatory on End of life care (Lancaster University). Researchers in relevant fields were contacted to access unobtainable articles found during the search process, and to obtain information on unpublished articles.

### Eligibility criteria

A list of inclusion criteria was applied to each screening stage (Table 1).

**Table 1:** Summary of eligibility criteria applied to studies

Inclusion Criteria	Exclusion Criteria
Studies written in the English language only	Studies written in languages other than English
Studies conducted in the U. K. and the Republic of Ireland (Ireland and Northern Ireland have an all-Ireland palliative care alliance).	Studies researching children’s hospices
Studies which include the perspective of family, patients and/families/informal caregiver	Studies which only have a focus on staff perspectives
	Studies only focusing on the clinical outcomes of treatments
The study was conducted within a dedicated hospice facility with other health care settings (i.e. care homes and hospitals), only used as a comparison	Studies focusing only on diagnostic elements of the illnesses of those in hospice care.
Studies researching adult hospices or hospice services only	Studies looking at only hospital palliative/end of life care
	Systematic reviews

### Screening of studies

After removing any duplicates, the remaining papers were independently screened by title and abstract to determine their eligibility for inclusion; as abstracts are often absent in grey literature, it was also necessary for titles, executive summaries and tables of contents to be screened. A random sample was taken by a second reviewer and the inclusion/exclusion criteria applied to check that the papers had been reliably kept or dismissed. After the initial screening stage, included studies were retrieved for full-text copies and read again to apply the inclusion/exclusion criteria; again, a sample was checked by a second reviewer to ensure that the inclusion/exclusion criteria had been applied accurately. Any disagreements were resolved through discussion.

### Quality appraisal

Four tools (Table 2) were used to assess methodological strengths and limitations of included studies, with those selected split into four categories: qualitative, quantitative, mixed-method and questionnaires/surveys. Method-specific tools were used to assess methodological limitations in primary studies and to guide the interpretation of the findings. Quality assessments were not used to exclude articles. A random sample of studies were chosen and checked by a second reviewer (See Additional file 2 for full quality appraisal). Disagreements were resolved by consensus.

**Table 2:** Quality appraisal tools

Study design	
Qualitative studies	Critical Appraisal Skills Programme (CASP) [4]
Quantitative studies	Effective Public Health Practice Project Quality Assessment Tool (EPHPP) [42]
Questionnaires and surveys	Centre for evidence-based management “critical appraisal of a survey” (CEBMa) [43]
Mixed- method studies	Mixed-method appraisal tool (MMAT) [44]

### Data extraction

Data extraction was performed using a bespoke form. The following domains were included: Title, author(*s*), publication date, study design, setting, objectives, data collection, sample characteristics and analysis methods. Qualitative evidence of interest were coded on the primary study. For the quantitative studies, findings were grouped by topic or outcome. Descriptive statistics, percentages, *p* values and estimates of precision such as confidence intervals were extracted. Author interpretations were also extracted. This table was then reviewed by a second reviewer. Only data relevant to the research question was extracted (See Additional file 3).

### Data synthesis

A mixed-method synthesis was utilised and conducted in three phases, whereby the studies were separated by design and synthesized sequentially, qualitative first, followed by quantitative and then an overarching synthesis.

#### **Phase 1:** Qualitative evidence

All studies exploring perspectives and views where value could be interpreted to generally indicate the implied value to patients and family-caregivers were synthesised using the Thomas and Harden [3] approach to thematic synthesis.

##### *Stage 1: Free line-by-line coding of textual findings from primary studies*

The process of coding and synthesising individual qualitative studies was completed manually, rather than using computer software packages such as NVIVO. This coding process involved the allocation of narrative codes to specific sentences which enabled data to be categorised. For this review, an inductive approach was utilised as codes were derived from the data itself.

##### *Stage 2: Organisation of free codes into ‘descriptive’ themes*

The second stage of the Thomas and Harden [3] approach involved the organisation of free codes into descriptive themes and, to increase the validity of the themes, regular collaboration with a second reviewer was undertaken until consensus was achieved.

##### *Stage 3: Generating* analytical *themes*

A defining feature of the final stage of the synthesis involves ‘going beyond’ the findings of the original data to yield ‘analytical themes’ which contribute to the creation of a synthesis that is more than just a description of the original studies [4]. With constant mindfulness of the review question, four analytical themes were inferred from the data (Additional file 4 demonstrates the transition from codes to analytical themes).

#### **Phase 2*:*** Quantitative evidence synthesis

It was not possible to undertake a meta-analysis as study designs, outcomes and measures varied. All quantitative studies where value was measured quantitatively were synthesised using a narrative summary approach. Findings were grouped by topic or outcome and summarised.

#### **Phase 3**: Cross study synthesis

The final stage involved the integration of the findings from both the qualitative and quantitative synthesis by juxtaposing data in a matrix (Table 3). This visual representation enabled the identification of new findings which went beyond the information gained from the separate synthesis of the quantitative and qualitative data. A table was created to map the values expressed by patients and carers across studies (See Additional file 5), followed by the creation of a matrix to integrate the comparable findings of the quantitative and qualitative synthesis. There was not a complete fit between the qualitative and quantitative evidence and the matrix represents where evidence on the same issue could be juxtaposed. Other qualitative findings that could not be mapped against comparable quantitative findings remain as standalone qualitative findings.

**Table 3:** Synthesis Matrix

Qualitative findings of what patients and carers valued	Quantitative findings of what patients and carers valued	What this means	Overarching finding
Availability of staff and access to out of hours for individuals receiving support from Hospice at Home to ensure that patients and carers had their physical and psychological needs met. Not everyone was able to access certain services associated with Hospice at Home.	Carers valued the support provided to them ensure patients’ wishes to stay at home were met. When compared to a hospital, hospice staff were mentioned more positively	Access to specialist staff and out of hours support was valued by patients and carers but was not always available to them.	*Equity in the provision of support is an essential value to ensure patients and their family caregivers are receiving timely interventions day or night*
Those nearing end of life valued a wide variety of diversional and therapeutic activities suitable to their changing needs and preferences	Patients valued a wide range of activities but patient satisfaction relating to the range of activities offered by the hospices has consistently declined over the years	Diversional and therapeutic activities were valued by people at the end of life, but hospices appear to be limiting the range and their availability.	*Choice and accessibility was a consistent value expressed by patients thus creating a need for a wide range of activities, especially at the end of life.*
Those closely affected by death valued that they were communicated with in a sensitive way and were offered immediate and ongoing bereavement, emotional and spiritual support.	Some carers felt abandoned by the hospice after the death of a loved one whilst others mentioned the benefits associated with a follow up call	Carers valued empathetic and appropriate bereavement care and follow up but not everyone received the same level of access to bereavement services and support	*Carers placed high value on bereavement support, but the reactive nature or lack services resulted in carers foregoing support*
Patients valued the provision of social opportunities, with many believing this had helped them retain some semblances of normality. Carers sometimes referred to the isolating nature of caring and some mentioned that they had taken advantages of ad hoc social opportunities (talking to other carers in shared rooms).	Family caregivers attended a bereavement support group to talk to someone outside their family	Patients and carers valued the social aspects of care and support but carers also need to be offered planned social opportunities.	*Caregivers valued the provision of social opportunities and could therefore benefit from access to official social support networks*
Continuity, accessibility and consistency in contact between patients, carers and key medical and social care professionals were clearly expressed as vital by both carers and patients	Carers identified that the lack of consistency in staff resulted in care providers who were unaware of the patients’ medical. This was especially prevalent within the Hospice at Home setting.	Patients and carers highly valued continuity of care but the standard of continuity varied and did not always meet expectations	*Equity in the provision of support is an essential value to ensure patients and their family caregivers are receiving timely interventions day or night*
Respite care offered valued breaks for carers which helped them retain a sense of normality and ensured they were able to continue their caring role but in some instances, respite could have been offered sooner.	Respite care was valued by carers across all settings and was a prominent reason for patient referral to day care	Respite care was highly valued but, in some instances, needed to be offered sooner.	*Carers placed high value on proactive support, but they did not always consistently receive it*
The provision of hospice staff night aides during times of crisis were of great importance to carers. Despite this, some carers described feelings of abandonment during times of need	A large proportion of carers were especially grateful for the ease at which they could access a wide variety of staff. No reference was made to a lack of necessary staff	The provision of staff who were able to support patients in their own homes at night were valued highly but their availability varied.	*Equity in the provision of support is an essential value to ensure patients and their family caregivers are receiving timely interventions day or night*

### Confidence in the synthesised findings

#### Qualitative findings

GRADE CERQual (Confidence in the Evidence from Reviews of Qualitative research) was used to assess and summarise the confidence in the review findings. The approach focuses on four components: (1) Methodological limitations; (2) Coherence of the review findings; (3) Adequacy of the data; (4) Relevance of the findings from the included studies to the review question. There are four levels of confidence that can be assigned to each finding; very low, low, moderate and high. All findings are initially classified as ‘high confidence’ and then demoted if important limitations were discovered across the four components (See Additional file 6).

#### Quantitative findings

GRADE CERQual is primarily designed for assessing the certainty of findings from trials. There is no GRADE equivalent for questionnaires/surveys so it was not possible to assess the confidence in the quantitative findings.

#### Reporting

The Enhancing Transparency in reporting the synthesis of qualitative research (ENTREQ) [5] was used for the qualitative evidence and relevant elements of the Preferred Reporting Items for Systematic Reviews (PRISMA) [6] was followed for the quantitative evidence.

## Results

**Fig. 1:**
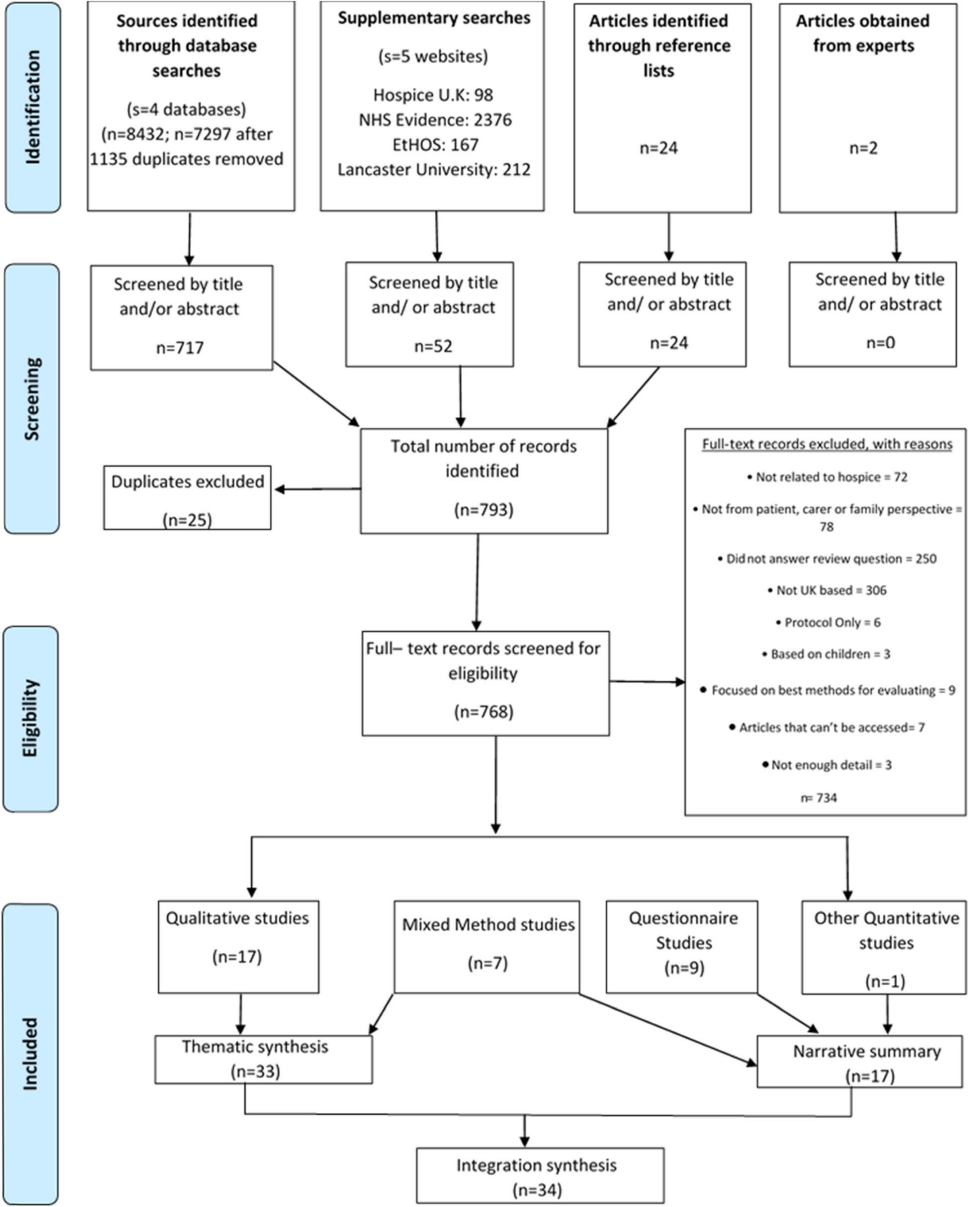
Flow chart on study selection process according to the PRISMA Guideline

A total of 34 studies were included (Fig. 1). An additional seven articles were not included as they could not be accessed (see Additional file 7). Attempts were made to access these articles via inter-library loans, Google searching and contacting the authors directly, however, despite all attempts, the seven articles could not be accessed.

### Qualitative findings

Four analytical themes demonstrating the value of palliative care and hospices services to patients and their family caregivers were developed from a thematic synthesis of 33 studies that reported qualitative findings. The themes were largely homogenous across studies and stakeholder groups (families/caregiver and patient) and the key findings are reported below. By way of illustration, specific values of the services from 15 studies were displayed in a table (See Additional file 5).

#### Analytical theme 1: The importance of staff in the provision of high standard quality care

The personal and professional traits of hospice personnel contributed greatly to the overall value attributed to hospice care. Patients and family-caregivers valued the personal qualities of staff, their experiences and specialised knowledge and skills, and development of a close rapport amongst staff, patients and their families. The importance attributed to these qualities was further evidenced with typical comments regularly referring to how staff had “*turned out to be friends”* [7]. The specialised knowledge and expertise of hospice personnel also resulted in the enhanced ability of staff to empathise, use their initiative, anticipate the changing needs of the patient and their families and provide proactive responses. This, however, would not have blossomed without continuity, specifically, regular contact with designated key personnel. Through regular and consistent contact, staff members were able to learn the small nuances of individual patients and their family-caregivers and provide the necessary support, tailored specifically to them. To illustrate, staff awareness of family-caregiver support needs ensured that respite care was often offered before families fatigued [7], preventing unwanted hospital admissions.

#### Analytical theme 2: The importance of the role of social engagement and participation in social activities in the maintenance of relationships and their sense of normality

Many patients expressed the value associated with a sense of community that was created in the day units by bringing people together who were in the ‘same boat’ [8, 9]. The hospice promoted a community ideology within which individuals were not judged on their actions and were given the freedom to sit and be accepted without feeling the need to contribute [10]. Frequent use of collective terms such as ‘we’ and ‘us’ only serve to strengthen this notion [10]. Day-care provided an environment which enabled patients to hold open discussions with each other about how their illness had affected their lives [8]. This was of value because it enabled patients to share their stories regarding their treatment, symptoms and personal experiences thus resulting in a reduction in their social isolation. Often patients would maintain a façade when in the presence of family and loved ones to protect them from greater emotional distress *(See Quote 4 in* Additional file 8*)*. Peer support proved to be the overwhelming value of day care.

#### Analytical theme 3: The importance of the comfort gained from the availability and accessibility of the hospice

Both family-caregivers and patients placed value on the availability and flexibility of the hospice services and its staff [9, 11–13]. Availability and flexibility was comprised of multiple facets, each of which has an individual value to service users. Patients and caregivers were quick to note that the availability of staff members [13], and the 24-h support they provided [11], coupled with other influential factors such as open visiting hours [14] and access to a wide range of staff and services, were central to a sense of security [7, 15]. In particular, the provision of phone support, addressment of worries, general support, reassurance, validation, and help with practical tasks had been a significant help to families in continuing with their caregiving role [16]. Many caregivers suggested they had willingly taken on the role in order to facilitate the patients’ wish to die at home [12]. This, however, had detrimental effects on their physical and psychological wellbeing largely due to the stress created in trying to care for their loved one [12, 17]. For this reason, significant value was placed on the provision of hospice night aides-hospice staff members who provided support to patients and family-caregivers through the night [11]. Although value was placed on the availability of daytime hospice aides, the presence of night aides, particularly in times of crisis, was reiterated throughout [16] *(Quote 11)*. The time immediately after death was often reflected on by family-caregivers as a period of difficulty due to the number of tasks that arose following a home death, such as arranging funerals, returning medical equipment and difficulties seeking bereavement support [13]. For caregivers of patients who died in a hospice inpatient setting, or supported by the Hospice at Home service, the burden associated with the aforementioned tasks, in many cases were alleviated by the hospice. Caregivers noted the value of this.

#### Analytical theme 4: The importance of the role of the hospice in helping promote patient and carer autonomy through the provision of various support mechanisms

Through the provision of both carer and patient support, hospices enabled patients to fulfil their wish to die at home, which was highly valued by patients [13]. The ability to fulfil the patients’ wish to die at home, however, was influenced by the carers’ ability to cope. It was evident that family-caregivers were often concerned that they would be unable to support the ever-changing needs of the patient [13]. Family-caregivers often associated a patients’ end-of-life experience with their own ability to address the needs of the patient [13]. The support provided by the Hospice at Home service was an invaluable source of support and reassurance during times where carers were struggling, which in turn helped foster patient autonomy (*Quote 14)* [18]. This support was provided through a range of mechanisms including but not limited to physical, psychological, social and financial help. Respite care, provided by the hospice enabled family-caregivers to have time to themselves during which they could relax and complete other day-to-day chores [11] and regain a sense of normality. It was apparent that the provision of domestic-related support was highly valued and, on occasion, it was noted that hospice night aides completed domestic chores on behalf of family-caregivers [18]. The benefit of this was twofold, not only ensuring that practical household activities were accomplished, but it also helped alleviate the burden that caring often entails [11]. During periods when carers felt unable to cope, knowing that scheduled visits were arranged gave them the confidence and determination to continue with their caring role [11].

### Quantitative findings

Quantitative data from 17 studies were collated in a narrative summary. Patient and family-caregiver values were grouped by topic or outcome.

#### The quality of care provided

The high standard of care provided by hospices was of great comfort and value to patients and caregivers. When compared with other health care service providers (home care, care homes and hospitals), the number of bereaved caregivers categorising the quality of care as excellent was highest when provided by a hospice setting [19]. This was further supported across this synthesis as carers consistently reported high levels of satisfaction (91–97%) regarding the quality of care hospices provided [20, 21]. Caregivers’ perceptions of quality were further ameliorated by the knowledgeable [20, 22], courteous and approachable staff [22]. These positive traits associated with members of the hospice team endowed both patients and caregivers with high levels of confidence in their capabilities [23–28]. Whilst findings suggest differences in the provision of care specifically the various health care providers (hospital, care home and home care), Parkes [29] found minimal discrepancies between hospice and hospital staff in relation to friendliness, approachability and helpfulness from spouses’ perspectives.

#### Availability of the hospice and its staff

The value associated with the availability and accessibility of the hospice and personnel were inferred by the emphasis placed on this facet of care with patients agreeing that they had access to an adequate amount of staff [20, 23–27]. Most caregivers felt that they could reach the hospice medical team when necessary and this was reflected by Lucas et al. [20] who found that 82% of carers had no difficulties obtaining medical support. In addition, 95% of carers felt that the Hospice at Home service was able to provide the help requested for their loved one [22]. This adds to the perception that staff availability is greater within a hospice, [28, 29] however, it is worth noting that less than 60% of respondents had received access to 24-h support [21]. In contrast, findings demonstrated substantial disparities associated with the availability of staff within the hospital settings. The disparities that exist between hospice and hospitals were further accentuated by Parkes [29], as spouses reported that they were more likely to talk to a wider range of staff whilst at a hospice. When asked “How many other members of the institution staff did you get to talk to?” 68% of participants at other hospitals said ‘none’ compared to only 15% at St Christopher’s (*P* < 0.002) [29].

#### Provision of information

Both patients and caregivers reported high levels of satisfaction pertaining to the receipt of adequate information whilst under hospice care [20, 28] as 90% of respondents felt that they had been kept suitably updated by Hospice at Home [20]. 75% of respondents felt that hospice doctors and nurses were able to explain the deceased person’s condition, treatment and tests in a clear and comprehensible way [20] whereas only 46% of respondents, by contrast, felt hospitals were able to do so [28]. Addington-Hall and O’Callaghan [28] noted that caregivers within their study were twice as likely to ‘always’ be kept informed within a hospice as opposed to a hospital setting (90% versus 44%).

#### Patient and carers views on their involvement in the care

Within the hospice day care setting, the percentage of those ‘very satisfied’ with their involvement in the planning of their care ranged from 57.3–70%, whilst in the inpatient setting this ranged from 66.8–71.2% [23–27]. This particular area, however, has fluctuated across the years as a reduction in the percentage of day care patients reporting the highest levels of satisfaction was shown [23–27].

Whilst hospices were shown to have involved carers in the shared decision-making process thus ensuring they were fully informed, hospitals waivered in comparison [28]. This was evidenced as findings highlighted how 11% of carers within the hospice setting compared with 21% within a hospital setting felt that that decisions had been made which their loved one would not have agreed with [28].

#### *Bereavement support*

Parkes [29] identified that no systematic attempt was made by their included hospice to support bereaved spouses. Some respondents, however, highlighted that they had been informally asked to remain in contact, an invitation accepted by just under a quarter of respondents [29]. This is in stark contrast to the findings in more recent studies which showed that the 81% of respondents received a follow up call as a minimum level of support [21, 30]. Other services varied from monthly memorial ceremonies which had high attendance rates (87%), a volunteer bereavement support service [30] and a bereavement information evening [21, 30]. Bereavement information evenings were evident in two studies, attendance at the bereavement information evenings were relatively low with an attendance of 33% at one hospice [30] and 11% attendance at the other hospice [21]. The reasoning behind this could be explained as a consequence associated with a lack of awareness as some of the respondents (28%) explained that they were unaware of the support networks available [21]. Bereavement support was also extended to patients in some cases [23–25]. In many instances, the percentage of patients who felt extremely supported rarely surpassed 50% [23–25]. This was a prominent issue within the day care setting [23].

#### The accessibility and quality of food

Within the day care setting at one hospice, the “welcome on arrival with tea and scones” was considered by many patients (61%) to be the most valued activity [18]. Lunch time itself was valued by half of the patients (50%) [18]. When asked about the quality of catering, the percentage of inpatients who considered the quality as excellent ranged between 65.1–72.7%. In the day care setting the percentage ranged between 69.4–72.7% [23–27]. Evidence also demonstrated that a large proportion of patients are happy with their access to food outside of set meal times (55.4–69.6%) [23–25]. Between 75 and 81% of carers within the survey conducted by the Office for National Statistics [19] believed that their loved one had received the necessary support needed to alleviate hunger and thirst. A small proportion (13%) of carers, however, felt strongly that the patient had not received adequate support to address these needs [19].

#### Respect and dignity

Addington-Hall and O’Callaghan [28] found that most carers (92%) believed hospice patients were ‘always’ treated with dignity within the hospice environment. This received further support from both the National Survey of Bereaved Carers [19] and McKay at al [21] who reported that most carers (97%) believed that patients’ dignity had been maintained. In contrast, only half of the respondents felt that the patients’ dignity was maintained in the hospital setting [28]. The percentage who felt they were always treated with respect in day care and inpatient ranged from 90.4–94.3% [23–27].

#### Symptom relief

Whilst Addington-Hall and O’Callaghan [28] found no significant difference (*p* < 0.01) in pain control measures between the hospice and hospital from the perspective of bereaved relatives, differences in the effectiveness of pain relief were noted. Carers were more than twice as likely to report that the patients’ pain had been relieved ‘completely all the time/ completely some of the time’ within the hospice setting opposed to a hospital [28]. The effectiveness of pain relief was a finding which was concurrent with other studies, as carers’ felt that the relief of symptoms far exceeded their expectations [21]. Similarly, Parkes [29] demonstrated how spouses at a hospice were less likely than those elsewhere to worry about a patients’ pain or its relief (9% vs 36% *p* < 0.05).

#### The provision of hospice transport

Questions relating to punctuality, comfort and safety of hospice transport were asked in surveys [23–27]. Across all the domains, the percentage of individuals who rated these areas as excellent always exceeded 55% [23–27]. Kernohan et al. [18] discovered that 38% of patients most valued their journey to the hospice and 31% felt that their journey home was the most valued activity.

#### Visiting hours

Open visiting arrangements were appreciated by both carers and patients [23–27] with carers taking the opportunity to visit the patient every day [29]. Some carers (53%) spent in excess of six hours a day visiting which was comparably higher than the time spent by carers within the hospital setting (9%) [29].

#### Respite

One reason for referral to day care was to provide respite to carers [18]. McKay et al. [21] demonstrated how respite care was found to be beneficial to a large proportion of carers (85%). Whilst Skillbeck et al. [17] determined that five showed improvements in their relative stress score, three demonstrated no change and four had a negative change in their scores post respite.

#### Social opportunities

The provision of social opportunities was of considerable value to both patients [18, 31] and carers [17] with the latter confirming that their social life had been considerably affected by their caring role [17]. The hospices helped to facilitate a “quiet time to chat” which was valued by more than half of the patients [18] with a further 42% citing the opportunity to meet with others in a similar situation as a reason for referral [18]. The opportunity to meet people was a recurrent finding as Goodwin et al. [31] found that just under half of respondents believed it to be the most valued outcome within day care.

### Cross study overarching synthesis

The overarching synthesis of qualitative and quantitative findings enabled identification of findings, which extend beyond the synthesis of the qualitative and quantitative data when analysed in isolation. Many of these findings were identified at the descriptive level (See Additional file 6). There was not a complete fit between the qualitative and quantitative findings and matrix (Table 3) represents where evidence on the same issue could be juxtaposed. Other qualitative findings that could not be mapped against comparable quantitative findings remain as standalone qualitative findings.

#### Overarching finding 1: Equity in the provision of support is an essential value to ensure patients and their family caregivers are receiving timely interventions day or night

Through the integration of both quantitative and qualitative evidence, the value of the Hospice at Home service is irrefutable. For instance, McLaughlin et al. [22] identified that most of the carers within their study believed that the Hospice at Home service had played a vital part in ensuring their loved one remained at home. This finding was further supported by Jack et al. [16] who found that several participants discussed how the service had prevented unwanted hospital admissions. There were, however, varying levels of satisfaction associated with some components of the Hospice at Home service. Whilst some participants left nothing but positive accounts relating to the support provided [16], others reported accounts of abandonment in times of need [12]. These conflicting accounts perhaps demonstrate the inequities of the available services, such as access to out-of-hours support due to geographic variations. A lack of awareness of the services provided by the hospice could also cause the inconsistencies in accounts [12].

#### Overarching finding 2: Carers appeared to place high value on bereavement support but the reactive nature of the service resulted in carers foregoing support

Carers placed high value on bereavement support but did not always receive it. The most common criticism evident within the literature associated with the bereavement needs of caregivers was the lack of contact from the hospice following the death of a family member [21]. Whilst some respondents felt that the support from the hospice ended abruptly after the passing of their loved one [29], evidence suggests that others were accessing post-bereavement support [30]. The domains of support evident within the included studies ranges from an initial follow-up call to monthly memorial ceremonies [30]. The proactive nature associated with the bereavement follow-up contact evident within some hospices resulted in a large proportion of respondents benefitting from the service [21, 29, 30]. This is in line with caregiver preferences as evidence demonstrates how carers value proactive contact from the hospice [30, 32]. This could also be considered as the minimum level of support necessary to ensure the gradual readjustment to a life without hospice involvement. This gradual adjustment could also be facilitated through the provision of pre- bereavement support whereby interventions delivered prior to death can help enhance the caregivers’ preparedness and acceptance [22].

#### Overarching finding 3: Carers appeared to place high value on proactive support but they did not always consistently receive it

The value associated with staff acknowledgement of carer needs was clear [7, 12, 16]. In some instances, the value that carers placed on respite care, was especially high during the terminal phase. Some evidence suggests, however that the provision of some support mechanisms were likely to be reactive as opposed to proactive, although not openly acknowledged by carers. This is reflected in the following exert: “the family was beginning to suffer from ‘sitting up’ [22]. This statement demonstrates how the family had already begun to feel the strain associated with caring for a loved one, particularly at night, before support was offered, thus demonstrating the reactive nature of the service.

#### Overarching finding 4: Choice was a consistent value to patients thus creating a need for a wide range of activities

It becomes apparent that patients place significant value on having access to a wide range of activities, however, since 2005 patient satisfaction seems to have dwindled [23–27]. Within the day-care setting specifically, patients highlighted that the least satisfactory area of service were the activities available to participate in [23]. This becomes a crucial finding as it was not just considered to be the sole reason for referral for some [18] but also one of the critical components of day-care as evidenced by the substantial research focus [18, 33, 34].

Throughout the included studies, reference is made regarding the need to consider the entire person and to meet their physical, emotional, spiritual and social needs. Whilst it is abundantly clear that a patients’ emotional and social needs are being adequately met, in reference to the physical needs, evidence does not go beyond the remit of the alleviation of physical symptoms. This shortcoming left some respondents indicating that they would like access to activities to help keep fit [18].

#### Overarching finding 5: Carers valued the provision of social opportunities and could therefore benefit from access to official social support networks

Whilst a large proportion of caregivers highlighted that the help they received had a positive influence on their ability to cope [7, 9, 11, 12, 16], there are notable areas for refinement and improvement, especially in relation to the availability of social support. Evidence suggests that caregivers were not accessing official social support networks prior to the death of their loved one [31]. Caregivers are under tremendous amounts of psychosocial pressures, with caregivers regularly discussing exasperated feelings of social isolation as a result of their role [7]. Whilst it is acknowledged that need for social support is often met through the social interaction and relationships with immediate or extended family, for some caregivers, the ability to converse with family members can be challenging [7]. These challenges can range from the difficulties derived from a patients’ physical condition to the altruistic nature of the caregiver themselves, where they do not wish to burden their loved ones [11]. The provision of a support network which extends beyond the family is seen to provide increased benefits [30]. This is further evidenced by Williams and Gardner [35] who demonstrated that caregivers would often take advantage of the social opportunities resulting from shared rooms.

## Discussion

This mixed study systematic review utilised patient and family experiences to infer values from the data which in turn helped to identify outcomes of care that are important to all those who benefit most from hospice services. Whilst the qualitative studies map onto some but not all of the quantitative findings, where possible, it was its integration which helped to not only further emphasise the importance of hospice care but also highlighted the discrepancies in accounts across studies. This in turn provided a more robust narrative and elucidates to further work which needs to be done to negate the disparities evident in care across regions and between different hospice settings.

Certain attributes of care were of more value depending on the hospice setting, specifically, social support for patients utilising the day-care units, 24-h support for families supported by the Hospice at Home service and pain and symptom management within the inpatient units. Despite this, the identification of shared priorities, that is what patients and families deemed valuable, remained relatively consistent across the literature despite some discrepancies which could be attributed to geographic variation. This suggests that there are pivotal attributes associated with a ‘good death’ irrespective of the setting. The concept of a ‘good death’, however, can be complex and highly individual therefore highlighting the importance of neglecting a ‘one-size-fits all’ approach to care in favour of a system which offers continuous holistic assessments in response to the changing needs of both the patient and their family.

The quality of pain and symptom management received frequent adoration from patients and caregivers however, perhaps surprisingly, it was the ability of hospices to deliver on the psychosocial domains of care which received consistently high praise. The social model of care associated with specialist palliative day care was considered one of the pinnacle domains of hospice care, where the importance of supporting the patients’ psychosocially were widely acknowledged. This was facilitated through the delivery of suitable activities specifically tailored to their abilities, the encouragement of communication through the provision of peer support and the development of friendships thus resulting in reduced feelings of isolation. In the context of this review, however, carers often expressed feelings of social isolation largely due to insufficient social support [17]. Findings demonstrated that carers frequently sought informal methods to address these needs however, such opportunities were scarce especially for those living in rural communities in North Wales [36]. When observing the wider literature, one can say with some degree of certainty that a social support network which understands the complexities associated with caring for an individual at the end-of-life would be beneficial [37].

Whilst it is beyond the purview of this review to demonstrate whether carers were receiving adequate social support from independent sources, the issue remains that the provision of social support within the hospice setting was lacking. This lack of support may be due to an inequity in services due to geographic variation which has been highlighted across varying domains within this review. It is safe to conclude that the provision of such services would only serve to compliment and strengthen that which carers are already receiving.

Variations in hospice services were further accentuated when discussing out of hour’s telephone support, a service primarily utilised by family-caregivers. Whilst some carers recounted how they felt abandoned due to the lack of 24-h telephone support, others recalled the great sense of comfort gained from knowing that this service existed, even if never utilised. Additionally, variations in the accessibility of the Hospice at Home service were evident. With home deaths often a patients preferred choice [19], the Hospice at Home service becomes a vital community resource. Whilst the End-of-life care implementation board acknowledges the importance of supporting patients to die at home, this notion is heavily reliant on the availability of families who often have no or very little experience of caring for someone nearing end-of-life. With some carers referencing the inequality of access to this specialist support, this demonstrates critical gaps in the availability which certainly is not a new criticism in the literature [38]. With the growth in the chronicity of certain malignant and non-malignant diseases, a greater demand on both palliative care services and carers will ensue and perhaps further accentuate these disparities in care. It is also important to highlight that the views and experiences of patients suffering from a non-malignant disease are underrepresented in both this review and in the wider literature [39] therefore their needs cannot be fully understood.

The physical and mental burden associated with the caregiver role has been shown to influence bereavement outcomes, outcomes which can be modifiable through the provision of suitable support [37]. Despite advisory bodies such as the National Institute for Health and Clinical Excellence (NICE) in the UK advocating for the offering of immediate and ongoing bereavement support to those who are closely affected by a death, contrasting accounts within this review suggest that in some instances, there is a lack of effective translation of policy in to practice. The universalistic approach to bereavement care whereby support is proactively offered to everyone was highly valued, however, evidently this approach was not adopted by all hospices. Inequities in accounts surrounding this provision of care could in part be a result of the hospices using different approaches. Alternatively, the inequities in accounts surrounding access to suitable bereavement support could also demonstrate the temporal nature of the evidence and how practice has changed over time. Therefore, what people want and value from hospice services seems to have evolved.

Finally, and perhaps unexpectedly, despite both patients and carers placing significant value on the support they received, there was a lack of evidence to demonstrate the importance of hospice volunteers in helping to deliver this support. As recent years have seen the boundaries of the volunteer role expanding, one can only speculate that the minimal evidence base within this review is a result of patients and family caregivers having difficulty distinguishing between those who are staff and those who are volunteers. This role development may in part be due to the recommendations put forth by a number of reports published in recent years such as that commissioned by Help the Hospices entitled ‘Volunteers vital to the future of hospice care’ [40] which formulated a number of recommendations concerning the future development of volunteers. This report was based on the premise that they are vital in ensuring that those who are accessing support from hospices are receiving a higher quality of care. Finally, congruent with the wider literature, this review further demonstrates how patients with a non-cancer diagnosis remain underrepresented in research.

### Strengths and limitations

In addition to the triangulation of qualitative, quantitative and mixed methods data, the strengths also stem from the explicit, rigorous and systematic approach. A comprehensive search strategy was created which was informed by an information scientist and utilised in the search of multiple relevant databases. Whilst grey literature was also included, there remains the possibility that potentially relevant papers were missed. The search itself was also restricted to include English language studies only. While this was justifiable as the decision was made to utilise only literature based in the U.K. and the Republic of Ireland (Ireland and Northern Ireland have an all-Ireland palliative care alliance), again, this could cause relevant papers to be missed. This will affect the generalisability of findings beyond the UK and Irish contexts. It is acknowledged that a rich literature base on palliative care exists outside the UK and papers which may have relevant findings to this study were excluded. A prime example is Steinhausers’s [41] seminal paper whose conclusions drew some parallels to the findings of this review. Another limitation lies in the screening of returned studies and the critical appraisal, as they were conducted independently with only a random sample selected to be cross examined by a second reviewer, however, a large proportion of studies were found to have had minor methodological limitations. Additionally, by using the CERQual approach to assess the confidence in the review findings, the synthesis of findings are more transparent. Further complexities were added when trying to extract patient and carer-specific data from studies which investigated both groups. As data was synthesised, it creates a possible risk that group specific values may have been overlooked. As most of the included studies were not designed to address the review question, the findings represent hypotheses and propositions as to what people value based on an interpretation of their experiences or attitudes or level of satisfaction.

## Conclusion

This is the first review to explore what patients and carers value from hospice care. Findings strengthen the existing evidence base and provide new insights beyond symptom management and health outcomes. Of particular importance was the social value placed on services that are only usually provided by hospices, such as highly individualised care (e.g. personalised catering), befriending, social support, meaningful occupation, and bereavement support. With large disparities in the availability of services, however, the underrepresentation of patients with non-malignant diseases and the limited evidence base demonstrating the adequate addressment of the social needs of carers, there continues to be considerable gaps that warrants further research. These findings are important for the further advancement of interventions and supportive services.

## Additional files

Additional file 1: Example search strategy for one database (DOC 35 kb)

Additional file 2: Full quality appraisal. (DOCX 27 kb)

Additional file 3: Included studies table (DOCX 36 kb)

Additional file 4: A table demonstrating the transition from codes to analytical themes (DOCX 22 kb)

Additional file 5: A table to demonstrate the breakdown of values across a selection of studies (DOCX 27 kb)

Additional file 6: CERQUAL, Confidence in qualitative findings table (DOCX 30 kb)

Additional file 7: A list of the seven articles that could not be accessed (DOCX 16 kb)

Additional file 8: Quotations from included studies and their corresponding theme. (DOCX 15 kb)

## Abbreviations

ASSIA: Applied Social Sciences Abstracts
CASP: Critical Appraisal Skills Programme
CEBMa: Centre for Evidence-based Management “critical appraisal of a survey”
CINAHL: Cumulative Index to Nursing and Allied Health Literature
ENTREQ: Enhancing Transparency in Reporting the Synthesis of Qualitative Research
EPHPP: Effective Public Health Practice Project Quality Assessment Tool
EtHOS: British Library e-these online
GRADE CERQUAL: Confidence in the Evidence from Reviews of Qualitative Research
MeSH: Medical subject headings
MMAT: Mixed-method appraisal tool
NICE: National Institute for Health and Clinical Excellence
U.K: United Kingdom
