# Supplementary material for: What do patients and family-caregivers value from hospice care? A systematic mixed studies review
Source: BMC Palliat Care. 2019 Feb 8;18:18. doi: 10.1186/s12904-019-0401-1 (PMC6368799; doi:10.1186/s12904-019-0401-1)
Supplement: Supplementary file 1 — Example search strategy for one database (DOC 35 kb) [file 12904_2019_401_MOESM1_ESM.doc]

| **Additional file 1:** Example search strategy | | | |
| --- | --- | --- | --- |
|  | **Hospice* AND Palliative AND** | | |
| **OR** | **Intervention / interest**  **(hospice care, palliative care, end of life care)** | **Perspective**  **(patients, families, carers)** | **Evaluation (quality of life, outcomes, quality of death, value)** |
|  | Care AND palliative OR end-of-life OR end of life OR terminal* OR respite | "caregiver*" OR "care giver*" OR "informal caregiver*" OR "informal carer*" OR "family caregiver*" OR "family carer*" OR "spousal carer*" OR "spousal caregiver*" OR carer* | “quality of life” or “QOL” or “QOD” or “quality of death” or quality or “satisfaction” or “comfort” or quality or “well-being” or “well being” or “experience*” or “preference*” or “value*” or “outcome*” or “perspective*” or “effectiveness”) |
|  | Home AND palliative OR end-of-life OR “end of life” OR terminal* OR respite OR "day care" | Family or “family member*” or families OR “spouse*” or “parent*” or “loved one*” |  |
|  | Service* AND palliative OR end-of-life OR "end of life" OR terminal* OR respite OR "day care" OR "inpatient unit" | “Patient*” or “service user*” or “client*” or “consumer*” or “care recipient*” |  |
|  | Support AND palliative OR (end-of-life OR "end of life") OR terminal* |  |  |
| Limiters | English Only and Humans only |  |  |
|  | | | 969 results |
