# Supplementary material for: What do patients and family-caregivers value from hospice care? A systematic mixed studies review
Source: BMC Palliat Care. 2019 Feb 8;18:18. doi: 10.1186/s12904-019-0401-1 (PMC6368799; doi:10.1186/s12904-019-0401-1)
Supplement: Supplementary file 2 — Full quality appraisal. (DOCX 27 kb) [file 12904_2019_401_MOESM2_ESM.docx]

| **Additional file 2:** Quality appraisal tools and outcomes | | | | | | | | | | |
| --- | --- | --- | --- | --- | --- | --- | --- | --- | --- | --- |
| ***Qualitative Study Appraisal Outcomes: Critical Appraisal Skills Programme (2017)*** | | | | | | | | | | |
|  | **Question** | | | | | | | | | |
| **Author** | **1** | **2** | **3** | **4** | **5** | **6** | **7** | **8** | **9** | **10** |
| Borland et al (2014) | Yes | Yes | Yes | Yes | Yes | Yes | Can’t Tell | Yes | Yes | High |
| Jack et al (2016) | Yes | Yes | Yes | Yes | Yes | No | Yes | Can’t tell | Yes | Medium |
| Jack et al (2014) | Yes | Yes | Yes | Yes | Yes | Yes | Yes | Can’t tell | Yes | Medium |
| Hopkinson and Hallett (2001) | Yes | Yes | Yes | Yes | Yes | No | No | No | Yes | Low |
| Hyde et al (2011) | Yes | Yes | Yes | Yes | Yes | Yes | Yes | Yes | Yes | Medium |
| Kennett (2000) | Yes | Yes | Yes | Can’t tell | Yes | No | No | No | Yes | Low |
| Kennett and Payne (2005) | Yes | Yes | Yes | Yes | Yes | No | No | No | Yes | Medium |
| Low et al (2005) | Yes | Yes | Yes | No | No | No | Yes | No | Yes | Medium |
| Williams and Gardener (2015) | Yes | Yes | Yes | Yes | Yes | No | No | No | Yes | Low |
| Hayle et al (2013) | Yes | Yes | Yes | Can’t tell | Yes | Yes | Yes | No | Yes | High |
| Gambles et al (2002) | Yes | Yes | No | Can’t tell | Can’t tell | No | No | No | Yes | Medium |
| Kirk (2002) | No | Can’t tell | Can’t tell | Can’t tell | Yes | No | Can’t Tell | No | Yes | Low |
| Woolf and Fisher (2015) | Yes | Yes | Yes | Can’t tell | Yes | Yes | Yes | Yes | Yes | Medium |
| Holdsworth (2015) | Yes | Yes | Yes | Yes | Yes | No | Yes | Yes | Yes | Low |
| Exley and Tyrer (2005) | Yes | Yes | Yes | Yes | Yes | No | Yes | No | Yes | Medium |
| Carlebach and Shucksmith (2010) | Yes | Yes | Yes | Yes | Yes | No | Can’t Tell | No | Yes | Low |
| Thomas (2001) | Yes | Yes | Yes | No | Yes | Yes | Yes | No | Yes | Low |

| ***Mixed- Method Study Appraisal Outcomes:*** *Mixed Methods Appraisal Tool (MMAT) (2011)* | | | | | | | | | | | | | | | | | | | | | |
| --- | --- | --- | --- | --- | --- | --- | --- | --- | --- | --- | --- | --- | --- | --- | --- | --- | --- | --- | --- | --- | --- |
| **Questions** | | | | | | | | | | | | | | | | | | | | | |
| **Author** | **Screening questions** | | **1.1** | **1.2** | **1.3** | **1.4** | **2.1** | **2.2** | **2.3** | **2.4** | **3.1** | **3.2** | **3.3** | **3.4** | **4.1** | **4.2** | **4.3** | **4.4** | **5.1** | **5.2** | **5.3** |
| Field et al (2007) | Yes | Yes | Yes | Yes | Yes | Yes | N/A | N/A | N/A | N/A | N/A | N/A | N/A | N/A | Can’t tell | Can’t tell | Yes | Yes | No | No | No |
| Kernohan et al (2007) | Yes | Yes | Yes | Yes | Yes | No | N/A | N/A | N/A | N/A | N/A | N/A | N/A | N/A | Yes | Yes | Yes | Yes | Yes | Yes | No |
| Goodwin et al (2002) | Yes | Yes | Yes | Yes | Yes | No | N/A | N/A | N/A | N/A | N/A | N/A | N/A | N/A | Yes | Yes | N/A | No | Can’t tell | Can’t tell | No |
| McKay et al (2013) | Yes | Yes | Yes | Yes | No | No | N/A | N/A | N/A | N/A | N/A | N/A | N/A | N/A | Can’t tell | Can’t tell | Yes | No | Yes | Yes | No |
| McLaughlin et al (2007) | Yes | Yes | Yes | Yes | Can’t Tell | No | N/A | N/A | N/A | N/A | N/A | N/A | N/A | N/A | Yes | Yes | Yes | No | Yes | Yes | No |
| Roberts and McGilloway, (2008) | No | Yes | Yes | Can’t tell | Yes | No | N/A | N/A | N/A | N/A | N/A | N/A | N/A | N/A | Yes | Yes | Yes | No | No | No | No |
| Skilbeck et al (2005) | No | Yes | Yes | Yes | Yes | No | N/A | N/A | N/A | N/A | N/A | N/A | N/A | N/A | Yes | Yes | Yes | No | Yes | Yes | No |

| ***Questionnaire Survey Appraisal Outcomes:*** *Center for Evidence-Based Management Critical Appraisal of a Survey (2017)* | | | | | | | | | | | | |
| --- | --- | --- | --- | --- | --- | --- | --- | --- | --- | --- | --- | --- |
| **Questions** | | | | | | | | | | | | |
| **Author** | **1** | **2** | **3** | **4** | **5** | **6** | **7** | **8** | **9** | **10** | **11** | **12** |
| Kernohan et al (2006) | Yes | Yes | Yes | Yes | Yes | No | Yes | No | No | No | Can’t Tell | Yes |
| Lucas et al (2008) | Yes | Yes | No | Can’t Tell | Yes | No | Yes | No | No | No | Can’t Tell | Yes |
| Hastie et al (2005) | No | Yes | Yes | Yes | Yes | No | Can’t Tell | Can’t Tell | No | No | Can’t Tell | Yes |
| Hastie et al (2007) | No | Yes | Yes | Yes | Yes | No | Can’t Tell | Can’t Tell | No | No | Can’t Tell | Yes |
| Hastie et al (2009) | No | Yes | Yes | Yes | Yes | No | Can’t Tell | Can’t Tell | No | No | Can’t Tell | Yes |
| Jenkins and Codling (2011) | No | Yes | Yes | Yes | Yes | No | Can’t Tell | Can’t Tell | No | No | Can’t Tell | Yes |
| Jenkins and Codling (2013) | No | Yes | Yes | Yes | Yes | No | Can’t Tell | Can’t Tell | No | No | Can’t Tell | Yes |
| Office of National Statistics (2015) | No | Yes | Yes | Yes | Yes | No | Can’t Tell | Can’t Tell | No | No | Can’t Tell | Yes |
| Addington-Hall and O’Callaghan (2009) | Yes | Yes | Yes | Yes | Can’t Tell | No | No | Can’t Tell | Yes | No | No | Yes |

| ***Quantitative Study Appraisal Outcomes :*** *Effective Public Health Practice Project (2017)* | | | | | | | | | | | | | |  |
| --- | --- | --- | --- | --- | --- | --- | --- | --- | --- | --- | --- | --- | --- | --- |
| **Questions** | | | | | | | | | | | | | |  |
|  | **A** | | | **B** | | **C** | | **D** | | **E** | | **F** | |  |
| **Author** | **1** | **2** | | **1** | **2** | **1** | **2** | **1** | **2** | **1** | **2** | **1** | **2** |  |
| Parkes (1979) | Can’t Tell | Can’t Tell | | Case Control | No | Can’t Tell | Can’t Tell | Can’t Tell | Can’t Tell | Can’t Tell | Can’t Tell | N/A | Can’t Tell |  |
|  | **Weak** | | **Moderate** | | | **Weak** | | **Moderate** | | **Weak** | | **Weak** | | **Global rating** |
| **Weak** |  |  |  |  |  |  |  |  |  |  |  |  |  |  |
