# Supplementary material for: What do patients and family-caregivers value from hospice care? A systematic mixed studies review
Source: BMC Palliat Care. 2019 Feb 8;18:18. doi: 10.1186/s12904-019-0401-1 (PMC6368799; doi:10.1186/s12904-019-0401-1)
Supplement: Supplementary file 3 — Included studies table (DOCX 36 kb) [file 12904_2019_401_MOESM3_ESM.docx]

| ***Additional file 3:***  *Included studies table* | | |  |  |  |  |
| --- | --- | --- | --- | --- | --- | --- |
| **Author and year** | **Purpose of the study** | **Study participants** | | **Study location/Setting** | **Study design (& method)** | **Main findings (significant results for quant findings)** |
| Addington-Hall and O’Callaghan (2009) | To compare hospice in-patientcare and hospital care from the perspectives of bereaved relatives. | 40 bereaved relatives (24 female, 15 male). | | Inpatient, London | Survey.  A comparative study using postal surveys | The provision of hospice care had a significant positive effect on patient involvement in decision making (p 0.09, 66% vs 46%) compared to the hospital comparator. Patients were also more likely within the hospice to agree that they had been adequately involved in the decisions about their treatment. There were statistically significant differences in reported pain control between hospice and hospital (p <0.01, 81% vs 39%). |
| Borland et al (2014) | To retrospectively explore the experiences of bereaved partners who had cared for a loved one living with a terminal illness in order to identify gaps in the literature. | A total of 7 caregivers (1 female, 6 male) participated in the study. The age of carers ranged from 50-68 years. | | Hospice at Home service, Northern Ireland. | Qualitative Interpretative research design. Semi-structured interviews were conducted with bereaved carers and analysed using thematic content analysis. | Key values identified included the need for support to help informal carers continue with their caring role. The development of a close relationship with staff who were deemed by carers to be experts ensured that they were able to understand carer needs and provide the necessary support and advice. |
| Carlebach and Shucksmith (2010) | To evaluate an out-of-hours service which was run out of a local hospice. | 6 patients, 8 carers, 4 district nurses, 6 Macmillan staff members, 2 General Practitioners, 1 out-of-hours emergency care service participated in the study. | | None specified, North East England. | Qualitative  Qualitative in-depth interviews. Thematic content analysis was utilised analyse the interview transcripts. | An out of hour’s telephone service providing both reactive and proactive support was heavily utilised by patients and carers. This service provided an informal support network which resulted in patients and carers feeling reassured and also helped patients to retain some semblance of independence. |
| Exley and Tyrer (2005) | To evaluate a pilot Hospice at Home scheme from the perspectives of bereaved carers | 12 carers (9 female, 3 male) were included in this study. Other demographic information not specified. | | Hospice at Home service, East Midlands. | Qualitative  Semi structured interviews analysed using a method informed by the framework approach (Ritchie and Spencer, 1994). | Carers were grateful that there was a service which helped alleviate the burdens of caring and ensure that their loved ones wishes to die at home were fulfilled. The quality of staff, the availability of services specifically respite care and access to pain medications and the necessary equipment was also deemed important. |
| Field et al (2007) | To explore the quality of adult bereavement support from the perspectives of bereaved people. | 105 bereaved family members and professional and volunteer bereavement workers | | Five hospice research sites, England. | Mixed method  A multi-method study. A national postal survey utilising both open and closed questions. Using SPSS, descriptive statistics were used to analyse the numerical data. Interviews and five in-depth organisational case studies were coded using thematic categories. | Bereaved carers valued the continuity between pre-bereavement and bereavement support. Four out of the five included hospices in phase 2 utilised volunteers in the delivery of bereavement support. 96% of the included organisation in phase 1 offered 1-2-1 support to bereaved people. It was noted that some hospices needed to better integrate their bereavement support. The delivery of bereavement support differed across the different hospices. |
| Gambles et al (2002) | To explore patient perception of reflexology treatments | 34 patients (33 female, 1 male) were included in this study, all participants had a cancer diagnosis (n=34). | | Outpatient department, North of England. | Qualitative  Semi structured questionnaires incorporating both open and closed questionnaires. Thematic analysis was used to analyse patient responses. | Reflexology was said to have provided both emotional and physical benefits. Falling under the purview of both categories, the opportunity to relax was the principle benefit for 91% of participants. By providing a service in a friendly atmosphere, a range of other positive outcomes ensued. Such outcomes included but were not limited to improved sleep, a reduction in anxiety and help with side effects. |
| Goodwin et al (2002) | To describe the services at five palliative day care from the patients’ perspective. | 102 patients (Response Rate (RR) 41%).  (50 Female, 52 male) participated in the study. Patient diagnosis was specified as either cancer (n=89) or non-cancer diagnosis (n=4). All patients age ranged from 35-87 years). | | Five palliative day care centres, London. | Mixed method  Semi-structured interviews were conducted with patients at a maximum of 3-time points. The third interview was only completed by 33% of patients. | Five hospices were the focus of the research and were subdivided into either social, medical/therapeutic or medical/social. Whilst there were changes between baseline interview and third interview and between different models of care, patient responses largely fell under three categories 1) meeting people, 2) getting out, 3) the place. By the third interview, there was a noticeable difference in responses from participants receiving support from the medical model. At baseline ‘support’ was the most important to 48% of participants in the medical model by third interview the theme ‘meeting people’ had become more prominent (30%). |
| Hastie et al (2005) | To seek the views of patients on the quality of the treatment and care provided. | 53 hospices distributed self-completion surveys to their patients. 2324 patients returned their survey (1398 day care users, 926 inpatients. | | 53 hospices from across the UK. List of specific locations not included. | Survey/questionnaire  The quantitative data from the surveys were entered and analysed using SPSS v12 whilst the qualitative (textual comments) were entered and analysed using Excel. | The availability of staff and volunteers at the hospice obtained many positive survey responses. Hospice staff helped to provide a service which encourage patient confidence in their work. The results indicate that staff worked hard to help ensure patients felt their privacy was respected as high levels of satisfaction were shown in the survey responses for both in-patients (88.1%) and day-care (89.8%). Patients also felt that their needs had been sufficiently met. Other areas which received high praise included cleanliness (83-88%), the general environment (84-85%) and involvement in the decision-making process with the opportunity to ask questions. Some of the weaker areas of the service was the availability of activities for service users. |
| Hastie et al (2007) | To seek the views of patients on the quality of the treatment and care provided. | 49 hospices distributed self-completion surveys to their patients. 1052 patients from the inpatient unit and 1352 day-care across the included hospices returned a valid survey. | | 49 hospices from across the UK. List of hospice locations not included. | Survey/questionnaire The quantitative data from the surveys were entered and analysed using SPSS v14 whilst the qualitative (textual comments) were entered and analysed using Excel. | Patient responses demonstrated high levels of satisfaction in many areas. Responses pertaining to staff in particular indicated that they were successful in helping to foster patient confidence and in ensuring patients were treated with respect. The availability of staff also achieved notable recognition as they worked to meet patients’ individual needs and wishes. However, positive responses were higher within the day care setting. Not unlike previous surveys, cleanliness and the general environment were rated highly. Survey responses also demonstrated that the level of support provided to patients when another group member died or was discharged was lacking as this received the lowest levels of satisfaction. The range of activities available to patients also identified as one of the weaker services provided, along with the availability of food outside meal times and patient involvement with care. |
| Hastie et al (2009) | To seek the views of patients on the quality of the treatment and care provided. | 52 hospices distributed self-completion surveys to their patients. 2222 patients returned their survey (1259 day care users (Response Rate (RR) 62%), 963 inpatients (Response Rate (RR) 41%). | | 52 hospices from across the UK (only England, Scotland and Ireland had participating hospices). | Survey/questionnaire The quantitative data was entered in SPSS and analysed, producing descriptive statistics. Textual comments were analysed separately using Microsoft Excel. | The hospice provided a service which helped reduce anxiety levels for patients after their first visit. The hospices excelled in many areas of care such as cleanliness, patient satisfaction with their involvement in their care and the opportunity to ask questions. However, it was the staff and the care provided which received overwhelmingly positive comments from patients. Patient confidence with staff was especially prominent in the day care setting. Irrespective of the many improvements, comments demonstrated that work was needed within the inpatient unit as some patients wanted time to make decisions about their care and individual needs where somewhat overlooked. Other areas where advancement needed was access to food for inpatients, the availability of activities and support in the event of a death or discharge of another patient. |
| Hayle et al (2013) | To evaluate the experiences of patients suffering from chronic obstructive pulmonary disease who also received support from specialist palliative care services. | A total of 8 patients (5 male, 3 female) participated in this study. The age ranged from 63-77 years).  Patients included in this study all had a primary diagnosis of chronic obstructive pulmonary disease. | | Inpatient and outpatient services, North West England. | Qualitative  Semi-structured interviews using a topic guide ensued. Hermeneutic phenomenological approach was utilised. | Palliative care for patients suffering with Chronic obstructive pulmonary disease was said to alter the patients physically, psychologically and spiritually. Changes also occurred in the patients preconceptions of hospice care. Physically patients improved as their symptoms were better managed resulting in a reduction in the number of hospital admissions. Psychologically, patients described that they felt a reduction in anxiety and stress. The changes to patients spiritually resulted in positive changes regarding death as patients were not as worried about the dying process as they had been previously. Additionally, by utilising a service which provided support to others, COPD patients explained how this gave them perspective. |
| Holdsworth (2015) | To describe the experiences of bereaved family carers and the role that care providers play in these experiences. | 45 informal caregivers (Response Rate (RR) 16.3%) (28 female, 17 male) participated in this study. The diagnosis of patients were categorised as either cancer (n=38) or non-cancer (n=6). | | Location not specified. | Qualitative  Forty-four 1-2-1 interviews were conducted (2 carers interviewed jointly). Interpretive thematic analysis using the Framework approach was utilised to analyse the interview transcripts. | The findings fell under six core themes 1) Social engagement, 2) care provider characteristics and actions, 3) carers ability, 4) preparation and awareness, 5) presentation of the patient at death, 6) after death support for protected grieving. The family-caregivers ability to maintain a sense of self was dependent on their ability to spend time with their family and the connections they were able to make with staff. Further to this, the provision of carer support ensured that patients wishing to be cared for at home were able to have their requests fulfilled. Family-caregivers also found it important to play an integral role in the decision-making process of the patient. However, carer involvement in decisions relating to symptom management was difficult. The balance between pain relief and sedation was difficult as carers wanted the patient to be comfortable but also able to communicate. It was therefore necessary to ensure that carers were fully aware of what to expect and were suitably prepared for the patients’ death. It was then essential to ensure that carers received the necessary support after the event. |
| Hopkinson and Hallett (2001) | To explore the experiences of patients whilst attending hospice day care in order to find out what is important to them. | 12 patients were interviewed (5 female, 7 male). Patients ranged from 50-86 years of age. All patients had some form of terminal cancer. | | Hospice day-care unit. | Mixed method  Using a phenomenological approach, 1-2-1 interviews were used to explore patient experiences of hospice day-care. | The findings of the study fell under four headings; 1) Feeling comfortable, 2) Feeling good, 3) Feeling less isolated, 4) Personal preferences. The hospice atmosphere ensured that patients were made to feel comfortable. The comforting nature of the hospice was further accentuated by the staff. Day care also helped patients’ self-worth by having the opportunity to make choices and their involvement in activities tailored to their abilities. Social isolation was an issue for many patients, this issue was said to have been alleviated through attendance at day care. Within this study, patients could be separated into those who “tolerating” their issue and those who were “adapting”. Those who were seen to be adapting valued having the opportunity to share stories, learn new skills and talk about their illness. Comparatively, those who were seen to be tolerating valued the distraction of day care. |
| Hyde at al (2011) | To explore service users experiences of palliative day care to understand what they value from the service. | 29 patients and 8 carers participated in the study. Patient diagnosis included cancer n=10, multiple sclerosis n=10, motor neurone disease n=3, other n=6). | | One hospice palliative care unit, England. | Qualitative  Descriptive, cross-sectional study. A semi-structured topic guide was used when conducting focus groups and 1-2-1 interviews utilising open-ended questions and analysed using the framework methodology (Richie and Spenser, 1994). | The findings from this study fell under four main themes; 1) The quality of staff, 2) Sense of community, 3) Relationships. The skills and expertise of staff were important to carers especially when it came time to handing over their caring responsibilities to the hospice. Carers also had the opportunity to obtain any advice which contributed to the overall positive experiences of day care. For patients, day care provided an opportunity for relationships and a sense of community developed. |
| Jack et al (2014) | To explore the perceptions and experiences of bereaved family carers’ receiving support from hospice at home services. | The participants in this study included 20 carers (15 Female, 5 male). Their family member was most likely to suffer from a cancer diagnosis (n=16). Age of carers ranged from 20-69 years). | | Hospice at Home services across the UK, North West England. | Qualitative  A qualitative study drawing upon a naturalistic interpretative approach. Semi structured interviews were conducted with carers and analysed using a thematic approach (Braun and Clarke, 2006). | End-of-life care provided to patients at home was shown to positively impact upon informal carers. The three themes that derived from the study were: (1) A valued presence (2) In good hands (3) Supporting normal life. The provision of support at home ensured that carers felt supported and great comfort was drawn from the presence of hospice staff. Carers were also able to obtain the necessary reassurance and validation from staff during scheduled visits. These scheduled visits ensured that carers were adequately supported and also provided carers with a small break which ensured that they could continue with their caring responsibilities. This service was said to help carers maintain a sense of normality. |
| Jack et al (2016) | To explore the perceptions of both patients and family caregivers in relation to the Hospice at Home service. | A total of 16 patients (Female n=10, Male n=6) and 25 family caregivers took part in the study. Patient diagnosis were split into cancer (n=12) or non-cancer (n=4). Patient age primarily ranged between 81-90 (n=11). | | Hospice at Home Service, North West England. | Qualitative  A prospective qualitative approach drawing upon a naturalistic interpretative design was utilised within this study. Using a topic guide, semi structured interviews ensued, and transcripts analysed using a thematic analysis approach (Braun and Clarke, 2006). | The findings within this study fell under two main themes: (1) Embracing Holism, (2) Service organisation. The four subthemes (1) Talking about, (2) Knowing and doing, (3) Caring for caregivers, (4) Promoting choice all contributed to the theme ‘Embracing Holism’ which was the main focus of the research. The provision of a hospice at home service meant that patients and caregivers were given choices. A service which enabled patients to die in the place of their choice and provides support to caregivers to help fulfil this wish was highly valued. The knowledge and expertise in addition to the personal qualities of staff ensured that relationships were formed. These relationships comforted both patients and carers and also meant that staff were able to engage patients and carers in difficult conversations about illness trajectories, death and dying. These relationships also provided a form of social stimulation resulting in improvements in psychological wellbeing. |
| Jenkins and Codling (2011) | To seek the views of patients on the quality of the treatment and care provided. | 39 hospices distributed self-completion surveys to their patients. 1984 patients returned their survey (1150 day care users (Response Rate (RR) 62%), 834 inpatients (Response Rate (RR) 45%). | | 39 hospices from across the UK (only England, Scotland and Ireland had participating hospices). | Survey/questionnaire The quantitative data was entered in SPSS Version 19 and analysed, producing descriptive statistics. Textual comments were analysed separately using Microsoft Excel. | Few disparities existed between inpatients and day care responses regarding their stay at the hospice as many commented on their high levels of satisfaction with the services provided. The general environment and cleanliness of the hospice were but two factors contributing towards the positive feedback expressed although the number of favourable responses were slightly higher within the inpatient settings. Contributing to the high-quality service, was the availability of staff and the ethos they created as they provided patients with respect, dignity and privacy. However, qualitative statements demonstrated the need for improvements within the inpatient setting to ensure privacy is maintained as issues of noise were a regularity. The ethos promoted by the hospice also ensured that day patients ended their first day with less anxiety then they arrived with. Nonetheless the day care unit was not without criticism, as the number of day patients who felt that they were adequately involved in their care did not surpass that of inpatients. Other suggested improvements included the need for a wider range of activities. The use of transport provided by the hospice was utilised by 70% of day patients, a service which was rated highly. |
| Jenkins and Codling (2013) | To seek the views of patients on the quality of the treatment and care provided. | 20 hospices distributed self-completion surveys to their patients. 1039 patients returned their survey (574 day care users (Response Rate (RR) 64%), 465 inpatients (Response Rate (RR) 50%). | | 39 hospices from across the UK (only England, Scotland and Ireland had participating hospices). | Mixed method  The quantitative data was entered in SPSS Version 21 and analysed, producing descriptive statistics. Textual comments were analysed separately using Microsoft Excel. | As with previous surveys, hospices excelled in areas relating to respect and dignity. In contrast to the previous year, patient satisfaction with privacy also received high praise also results demonstrated an increase in both the day care and inpatient units. These improvements were seen across other areas such as advanced care planning, the variety of activities available, confidence in staff and hospice cleanliness. These were but few improvements. For day care patients, higher percentages of those demonstrating a reduction in levels of anxiety after their first visit were evident. However, improvements were not noted in all areas as satisfaction with hospice transport had decreased. Qualitative statements were able to clarify issues with pick up times, certain drivers and comfort. Specific to inpatients, improvements were seen in the following areas: disturbance from noise, food quality and visiting arrangements. |
| Kennett (2000) | To understand the experiences of terminally ill patients who were participating in art exhibition. | This study included 10 patients (6 male, 4 female). The majority of patients had a cancer diagnosis (n-8) compared with having a non-cancer diagnosis (n=2). Age ranged from 23-80 years. 11 members of staff were also interviewed. | | A palliative care day centre at one London hospice (St Christopher’s). | Qualitative  A phenomenological study. In depth semi-structured interviews were conducted with patients on a one to one basis whilst staff interviews were conducted in pairs. The interviews were transcribed and analysed to identify themes. | The themes that developed within this primary study demonstrate the positive outcomes of hospice day care on patients. (1) Enjoyment, (2) enthusiasm, (3) excitement, (4) pride, (5) achievement, (6) satisfaction, (7) sense of purpose, (8) mutual support, (9) permanence. Hope and a sense of purpose and encouragement through mutual support were the overwhelming outcomes within this study. These feelings were encouraged through the creation of a mural and the development of new skills. Some patients commented on how these positively affected patient symptoms. |
| Kennett and Payne (2005) | To analyse patients’ accounts of how living with a terminal illness affects their quality of life and experiences of attending St. Christopher’s Hospice. | This study included 34 patients (female n=11, Male n=23). Age range between 40-95 years. | | A palliative care day centre at one London hospice (St Christopher’s). | Qualitative  34 patients took part in six audiotaped recorded "goldfish bowl" teaching sessions. The data was analysed to identify themes | Patient responses were relatively consistent. The principle issues derived within this study focused how the patients illness affected their quality of life and their relationships with their families, their interactions with healthcare professionals and the experiences of attending the Creative living centre. Patients often discussed the difficulties transitioning to a life with a terminal or life limiting illness and the ultimate loss of their independence. Whilst some patients recounted feelings of anxiousness before their first visit but noted a change in mood over time. Other positive outcomes for patients included a sense of achievement, a feeling of belonging due to the mutual support and a chance to talk about their illness with others. |
| Kernohan et al (2006) | To explore and describe patient perspective on the provision of medical, social and therapeutic out-patient services | 26 patients participated in the study (10 male, 16 female).  All patients ranged from 31-70 years and were diagnosed with a form of cancer. | | A day care centre at one hospice in Northern Ireland | Survey/questionnaire  Semi-structured questionnaires were completed by 26 of the 50 day care patients attending one day care centre. The responses to closed questions were entered into SPSS for basic descriptive analysis whilst qualitative statements were subject to content analysis. | This paper explored patient satisfaction with hospice day care. The most common reason for patient attendance at hospice day care was to obtain emotional/spiritual support (61%), followed by the opportunity for families to have some respite (42%) and to meet other patients (42%). These opportunities provided significant benefits and resulted in positive outcomes such as reduced isolation and the development of relationships. Day care also provided the opportunity to get out. From a predetermined list, the most valued aspect of hospice day care that were valued the most included welcome on arrival with tea (n=16) followed by the provision of quiet time to chat with others. The facilitation of group discussions was valued by 58% of patients. Whilst most respondents felt that the hospice provided enough activities, 27% suggested the need for more craft orientated activities. Most patients (69%) found comfort in knowing that the hospice could meet their changing needs, facilitated by the availability of medical staff. |
| Kernohan et al (2007) | To access the spiritual needs of patients and their interactions with the hospice chaplains against the national standards for Hospice and Palliative care Chaplaincy | 62 patients (Response Rate (RR) 64%) participated in the study (31 female, 31 male).  All patients were 41 years of age or older. Patient diagnosis was not specified, however, it was explained that “most” had been diagnosed with cancer. | | Hospice day-care services, community care and Hospice inpatient unit in Northern Ireland. | Surveys/Questionnaire  Two methods of data collection were utilised. The first method involved reviewing participant pastoral notes followed by semi-structured interviews utilising both open and closed questions. The two data sources were linked and analysed using SPSS Version 11.5 to obtain descriptive statistics. Content analysis was used on the data generated from the open-ended questions. | With a high proportion of participants having religious beliefs (92%) access to spiritual support was found to be helpful to 90% of patients with the frequency of visits found to be appropriate for 52% however, 43% found that the visits needed to be more frequent. This service encouraged feelings of hope, helped patients prepare for death and reassured patients that their families were supported. Additionally, the chaplaincy service helped facilitate patient communication needs. |
| Kirk (2002) | This study explored patient preferences of single or shared rooms within the hospice. | 24 patients (12 female, 12 male) participated in this study. | | Hospice inpatient. Specific location not specified. | Qualitative  1-2-1 structured interviews were conducted with patients. Mode of analysis not specified. | The opportunity to stay in a single room was favoured over staying in a shared room by 75% with many patients previously having experiences of staying in a single room (96%). Privacy was the principle reason for wanting to stay in a single room, closely followed by the quiet nature associated with a single room, and to reduce embarrassment of symptoms. |
| Low et al (2005) | To explore the experiences of patients utilising palliative day care services. | The sample in this study included 18 patients, 12 carers, 11 palliative care day service managers and 22 volunteers. | | Palliative care day services across the UK. | Qualitative  A qualitative methodology was utilised. A total of four focus groups were facilitated by the research nurse and the Senior Research Fellow. The focus groups were transcribed verbatim for thematic content analysis. | Access to palliative day care professionals situated in one location was important to patients, where they could monitor patients regularly and pre-empt further health issues. The reasons behind admission to day care was often for either social or medical benefits. The overwhelming social benefit associated with admission was peer support where a reduction in isolation was felt and provided a service which patients looked forward to attending. Some patients acknowledged that they attended day care for improvements in physical functioning and mobility.  Day care also provided carers with respite resulting in improved quality of life. The medical knowledge of staff members ensured that carers could confidently leave patients at day care. Patients were disappointed when they had to be discharged. |
| Lucas et al (2008) | The aim of the study was to evaluate the Bradford hospice at home service through the exploration of carers, nurses and General Practitioners perspectives. | This study included carers (n=289) (Response rate (RR) 50.7%), district nurses (n=508) and GP’s (n=44).  Participant characteristics not specified. | | Hospice at Home service, Bradford. | Survey/Questionnaire Postal questionnaires were sent to carers.  The Quantitative data was analysed using SPSS frequency analysis. Qualitative data was analysed using grounded theory techniques. | The hospice at home service provided accessible support to carers which ensured that they were able to fulfil patients wish to die at home. This service was valued by 97% of carers who felt that the hospice staff were knowledgeable about patient conditions (92%). Whilst 80% of participants felt that there was sufficient nursing help, negative comments about the use of agency staff were said in abundance. |
| McKay et al (2013) | To examine whether a model established in one hospice helped to mitigate carer burden. | 122 carers (17 bereaved, 81 current carers) returned questionnaires.  29 carers agreed to participate in qualitative interviews.  The age of participants ranged from 25-85+. | | A Hospice at Home service. Midwest Ireland. | Mixed method design that included a postal survey and interviews. Different versions were given to current carers and bereaved carers.  Questionnaire data was analysed using SPSS 18.0. The qualitative responses were coded and thematically analysed. | The hospice at home service provided support to both patients and carers and 96% of respondents felt that referral to the hospice at home service had happened at the right time, however knowledge regarding some services was lacking. Access to 24-hour support and night time visits often provided a form of respite for carers. Respite was valued by 83% of carers and support at night was valued by 74%. Accessibility of the team was noted by 77% of carers. 75% of patients and 69% of carers felt communication, specifically staff sensitivity and continuity of care were good. Despite the positives expressed about 24-hour support and open communication, these failed to match or exceed respondent expectations. With regards to place of death, there was a statistically significant correlation between those who had discussed place of death with staff and the location in which the patient had died. After the patients’ death, 72% of carers had contact with the hospice at home team and 11% chose to attend a bereavement support service. 53% of carers also indicated that they would have liked to take a course to optimise their caring skills. |
| McLaughlin et al (2007) | To explore bereaved caregivers’ experiences of Hospice at Home | 128 family carers (Response Rate (RR) 41%) responded to the questionnaire.  (Demographic details not provided). | | Hospice at Home setting. Location not specified. | Mixed method  Postal Questionnaire made up of primarily closed questions and one open ended question. Quantitative data was analysed using SPSS, windows 11. Descriptive analysis was performed on all closed variables. Content analysis was performed on the qualitative data. | The findings within this study fell under five headings: (1) Awareness of service, (2) Home care, (3) Help requested, (4) Relieving carer burden, (5) Hospice at home staff. Whilst 94% of carers were aware of the involvement of hospice at home staff in their care, the qualitative comments revealed a lack of awareness regarding the hospice at home service. Whilst reasons behind why carers chose to care for patients differed, 95% felt that the hospice at home service ensured that carers could continue to support a patients’ wish to die at home, which was the most valued aspect of the service. Such support mechanisms included a night sitting service, carer relief reassurance and skilled services. Some carers did note that they would have liked more support. On the contrary, 95% of patients agreed that the service was available to provide the requested help such as accessing equipment and relieve the burden placed on carers. The courteous and approachable nature of the hospice personnel were said to have positively affected carer experiences (98%). Ninety-four percent of carers were said to hold enough knowledge regarding patient conditions however, qualitative statements suggest more training was needed for patients who were cognitively impaired. Suggested improvements included more support after bereavement. |
| Office for National Statistics (2015) | To collect information on the views on the quality of care provided to a friend or relative in the last 3 months of life from the perspective of bereaved people. | A sample of approximately 49,000 adults in England. Other information not specified. | | Postal survey | Survey/questionnaire Analysis was conducted using various statistical packages (None specified). | By looking at the views on the quality of care provided to a friend on relative in the last 3 months of life, it was discovered that 75% rated the overall quality of end of life care as excellent, outstanding or good across all care settings. However, it was the hospices who excelled in many areas, specifically, in relation to pain, dignity and respect. Healthcare personnel at hospices were said to excel in ‘always’ treating patients with dignity compared with any other care setting (care home, home care and hospitals). Of note, is the disparities between cancer patients and patients with Cardiovascular diseases as cancer patients were more likely to agree that they had been treated with dignity. Additionally, pain was said to have been relieved “completely, all of the time” more frequently than other healthcare providers. Co-ordination of care within the hospice could improve as it was discovered that the co-ordination of care within the home setting exceeded all other health care provider settings included in the study (hospice,hospital,care homes). |
| Parkes (1979) | To evaluate in-patient services at St Christopher’s Hospice | 34 bereaved spouses (22 female, 12 male). Demographic and diagnosis not specified. | | St Christopher’s Hospice. ‘Other’ hospitals unknown. | Quantitative  Self-assessments of 34 spouses of patients at one hospice were compared with the self-assessments of a matched group of spouses of patients who had died in a hospital. Data analysis not specified. | An evaluation of St Christopher’s hospice discovered that the number of spouses spending 6 or more hours at the hospice exceeded that of spouses visiting patients at other hospitals. Other differences between the hospice and hospital included the increased likelihood that spouses from the hospice would see a doctor compared to spouses at another hospital. Significant differences were evident in the perceived busyness of staff as staff at the hospice were less likely to be considered busy (Ward nurses p=<0.01; ward sister p=<0.05; ward doctor, <0.01). The support provided by both the hospice and the hospital were reported to have reduced anxiety for spouses compared to when patients were cared for at home however, spouses at a hospital were more likely to have anxiety symptoms (p<0.05). Specifically, loss of appetite was greater within a hospital setting (p<0.05) and the level of worry regarding patient pain. There were no support systems in place to support spouses during bereavement. |
| Roberts and McGilloway (2008) | To evaluate a hospice-based  bereavement support service in Ireland | 243 carers participated in this study. (Demographic details not stated). | | A hospice located in Ireland. | Mixed method  A cross-sectional postal survey. Carers completed: 1) a Bereavement Services Questionnaire;  2) a measure of grief reaction (TRIG) and 3) a measure of religiosity (SCSORF). | The focus of the study evaluated the utilisation of three bereavement support services. The services, a bereavement follow up service which had contacted 84% of respondents, a monthly memorial ceremony attended by 87% of respondents, a bereavement information service attended by 33% of respondents and a volunteer bereavement support service. A number of reasons were given as to why some respondents did not attend services. Additionally, respondents who had attended a bereavement service demonstrated higher levels of grief symptoms. |
| Skilbeck et al (2005) | To explore carer experiences  and expectations of respite. | 25 patient/carer dyads recruited (17 male patients, 8 female). Carer gender not reported in detail. Age ranged between 36-88 years. Diagnosis was separated into cancer (n=15) and neurological conditions (n=12). | | Independent hospice providing inpatient respite beds. North of England. | Mixed method.  Prospective study using mixed methods data collection. The first method utilised  Semi-structured interviews at 2 time-points. The additional method used was the Relative Stress Scale Inventory which was administered at both interviews conducted with carers. The qualitative data was analysed using constant comparison. Categorical data from the structured questionnaire were entered in to SPSS and descriptive and comparative statistical analysis ensued. | The physical, and psychologically demanding nature associated with caring for a loved one was noted. The Relative Stress Scale inventory (RSSI) indicated that providing care for someone else had a considerable impact on their lives. Whilst many carers (76%) believed that respite had met their expectations, the RRSI indicated that only four carers had received a positive change in their score post respite whilst 3 carers demonstrated no change and for 5 carers, there was a negative change in scores. Irrespective of this, qualitative statements demonstrated that a majority of carers felt that respite had held been beneficial with only a small proportion expressing dissatisfaction with the service, specifically a lack of awareness and the frequency at which respite occurred. |
| Thomas (2001) | Patient preferences were explored regarding visiting | 6 patients (Demographic information not specified). | | Inpatient setting. Location not specified. | Qualitative  Phenomenological approach. Semi-structured interviews. Thematic analysis was utilised. | Opening visiting hours was shown to bring many benefits to patient. In particular, some patients felt visits helped them to cope by enabling them to maintain connections and gain support. However, some patients felt that open visiting hours inhibited them as they explained how they felt that they were not in control. This lack of control often negatively affected a patients’ ability to cope. |
| Williams and Gardiner (2015) | To understand the preferences of patients, family and staff for single or shared rooms within the hospice. | 29 patients (8 female, 6 male), 23 carers (16 female, 7 male) and 10 hospice staff participated in this study. Age of participants ranged from 44-89. Information on diagnosis not stated. | | Inpatient and day care patients, and staff from one hospice in Sheffield. | Qualitative  Semi-structured interviews following an interview schedule. Audio recordings were not taken therefore hand-written notes were made and analysed using thematic content analysis. | The findings of this study determined that patients and carers should be offered the choice of single or shared rooms. The social aspects that derive from a shared room were the principle benefit of shared room. However, the lack of privacy was a consequence of a shared room, due to this many patients and carers preferred the single room alternative. |
| Woolf and Fisher (2015) | To explore patient experiences of dance movement psychotherapy in day care. | 4 patients (Female (n=2), Male n=2). Age of participants ranged from 82-95 years.  Diagnosis varied (cancer, cardiac disease, chronic obstructive pulmonary disease, diabetes, and rheumatoid and osteoarthritis. | | Day hospice. Location not specified | Qualitative  A case study approach triangulating both verbal and non-verbal data analysed using thematic analysis following modified phenomenological techniques. | Dance movement psychotherapy (DMP) was a service able to positively effect patients psychically, emotionally and socially. The conditions of the service ensured that patients felt safe, and relaxed. |
