# Supplementary material for: What do patients and family-caregivers value from hospice care? A systematic mixed studies review
Source: BMC Palliat Care. 2019 Feb 8;18:18. doi: 10.1186/s12904-019-0401-1 (PMC6368799; doi:10.1186/s12904-019-0401-1)
Supplement: Supplementary file 4 — A table demonstrating the transition from codes to analytical themes (DOCX 22 kb) [file 12904_2019_401_MOESM4_ESM.docx]

| **Additional file 4:** *Table demonstrating the transition from codes to analytical themes* | | |
| --- | --- | --- |
| Analytical theme  What people valued | **Descriptive themes** | **Codes** |
| The importance of highly skilled staff in the provision of high standard quality care | 1. The value of highly skilled and attentive staff members to patient and family-caregivers 2. The comfort gained from the development of good relationships with health-care professionals 3. The importance of staff awareness in regards to patient and family-caregiver needs 4. Continuity of care was deemed important | 1. Regular monitoring 2. Consistency of staff 3. Inter and intra agency co-operation 4. High standard of nursing and medical care 5. Awareness of patient condition 6. Attentive staff 7. Going above and beyond 8. Specialised expertise and knowledge 9. Awareness of patient and family-caregivers needs 10. Designated Key professionals |
| The important role of social engagement and participation in social activities in the maintenance of relationships and their sense of normality | 1. Social opportunities helped to develop important relationships with other patients and family-caregivers 2. Help to develop old and new skills 3. Social opportunities helped to maintain a sense of normality 4. Peer support provided a support network | 1. Something to look forward to 2. Welcomed distraction 3. Acceptance and understanding 4. Peer support 5. Learning new skills 6. Developing friendships 7. Meeting new people 8. Communication 9. Sharing experiences |
| The importance of comfort gained from the availability and accessibility of the hospice | 1. Access to a wide range of services and staff for patients and family-caregivers 2. Availability of the hospice 3. Hospice atmosphere ensured patient and family-caregiver comfort | 1. Hospice transport 2. Therapeutic environment 3. Feels like home 4. Respite care 5. Availability of staff 6. 24-hour support 7. Open visiting hours 8. Phone support 9. Availability in a crisis 10. Time with staff 11. Night aides 12. Access to a range of services 13. Provision of medical equipment |
| The important role of the hospice in helping promote patient and carer autonomy through the provision of various support mechanisms | 1. Maintenance of psychological, spiritual and physical well- being 2. Promoting patient and carer independence through choice 3. Practical support for patient and family-caregivers | 1. Support to stay at home 2. Being listened to 3. Practical support 4. Signposting to other agencies 5. Clinical information and advice 6. Improved psychological well-being 7. Improved physical well being 8. Preventing unwanted hospital admissions |
