# Supplementary material for: What do patients and family-caregivers value from hospice care? A systematic mixed studies review
Source: BMC Palliat Care. 2019 Feb 8;18:18. doi: 10.1186/s12904-019-0401-1 (PMC6368799; doi:10.1186/s12904-019-0401-1)
Supplement: Supplementary file 5 — A table to demonstrate the breakdown of values across a selection of studies (DOCX 27 kb) [file 12904_2019_401_MOESM5_ESM.docx]

| **Additional file 5:** *A breakdown of values across a random selection of 15 studies*. |  | |  |  |  |  |  |  |  |  |  |  |  |  |  |
| --- | --- | --- | --- | --- | --- | --- | --- | --- | --- | --- | --- | --- | --- | --- | --- |
| **What do patient and carers value from hospice services?** | Borland et al, 2014 | Carlebach and Shucksmith, 2010 | Exley and Tyrer, 2005 | Gambles et al, 2002 | Hayle et al, 2013 | Holdsworth, 2015 | Hopkinson and Hallett, 2001 | Hyde et al, 2011 | Jack et al, 2014 | Jack et al, 2016 | Kennett, 2000 | Low et al, 2005 | Lucas et al, 2008 | McKay et al, 2013 | McLaughlin et al, 2007 |
| Availability and accessibility of the hospice services and staff were a source of reassurance especially out of hours support. | 🗶 | ⚫🗶 | 🗶 |  |  | ⚫🗶 | ⚫ |  | 🗶 |  |  | ⚫ | 🗶 | 🗶 | 🗶 |
| - Both patients and carers benefit from the personalities, expertise and specialised skills of staff | 🗶 |  | 🗶 | ⚫ | ⚫ | ⚫🗶 | ⚫ | ⚫🗶 | 🗶 | ⚫🗶 |  | ⚫🗶 | 🗶 | 🗶 | 🗶 |
| Patients and carers valued the opportunity to develop meaningful relationships with staff | 🗶 |  |  |  |  | ⚫🗶 |  |  |  | ⚫🗶 |  |  |  |  |  |
| Patient needs were always met by staff however, it was of value that staff members were also aware of carer support and health needs. | 🗶 |  | 🗶 |  |  |  |  |  |  | ⚫🗶 |  |  |  |  | 🗶 |
| Continuity of care ensured that patients were regularly monitored, staff were consistent and therefore were aware of the patients’ individual health needs, good intra-and inter agency cooperation and pre-and-post bereavement support. |  | ⚫🗶 | 🗶 |  | ⚫ | ⚫🗶 |  | ⚫🗶 |  |  |  | ⚫ | 🗶 | 🗶 |  |
| The provision of social opportunities enabled patients to talk to others who they consider to understand what they are going through and helped reduce isolation as meaningful friendships developed | 🗶 |  |  |  | ⚫ | ⚫🗶 | ⚫ | ⚫ |  | ⚫🗶 | ⚫ | ⚫🗶 |  |  |  |
| The ability to maintain a sense of normality was important. |  |  |  | ⚫ |  |  | ⚫ |  | 🗶 |  |  |  |  |  |  |
| Timely access to a wide range of staff, services and activities | 🗶 | ⚫🗶 |  | ⚫ |  |  | ⚫ |  |  |  | ⚫ | ⚫ | 🗶 | 🗶 | 🗶 |
| Time spent with staff was especially important as it ensured that patients felt that they were being listened to. |  |  |  | ⚫ | ⚫ |  | ⚫ | ⚫🗶 | 🗶 |  |  |  |  | 🗶 |  |
| Hospice atmosphere encouraged a sense of comfort and provided a homely feel. |  |  |  | ⚫ | ⚫ |  | ⚫ |  |  |  |  |  |  |  |  |
| Support to maintain psychological, spiritual and emotional well- being | 🗶 |  |  | ⚫ | ⚫ |  | ⚫ | ⚫ |  | ⚫🗶 | ⚫ |  |  | 🗶 |  |
| Symptom management | 🗶 |  | 🗶 | ⚫ | ⚫ | ⚫🗶 |  |  |  | ⚫🗶 |  |  | 🗶 | 🗶 |  |
| Promoting patient and carer choice by ensuring their priorities and choices were at the forefront of end of life care planning. |  |  |  |  |  | 🗶 | ⚫ | ⚫ | 🗶 | ⚫🗶 | ⚫ |  |  | 🗶 |  |
| Practical support for patients and carers, including financial and domestic support and signposting to other agencies | 🗶 |  | 🗶 |  |  |  |  |  | 🗶 | ⚫🗶 |  |  |  | 🗶 | 🗶 |
| Being prepared for death, knowing what to expect as the illness progresses and having access to bereavement support when needed. This is often facilitated through honest conversations. | 🗶 |  |  |  |  | 🗶 |  | 🗶⚫ | 🗶 | ⚫🗶 |  |  | 🗶 | 🗶 | 🗶 |
| The provision of clinical information and advice, the opportunity to ask questions and obtain reassurance. | 🗶 |  |  | ⚫ |  | 🗶 |  | ⚫ | 🗶 |  |  | 🗶 |  | 🗶 | 🗶 |
| Respite care to allow valued breaks for carers | 🗶 |  | 🗶 |  |  |  |  |  | 🗶 | 🗶 |  | 🗶 |  | 🗶 | 🗶 |
| Being treated respectfully |  |  |  |  |  |  |  |  |  |  |  |  | 🗶 |  | 🗶 |
| Being able to share ideas and experiences with others in similar situations who understand what they are going through |  |  |  |  |  |  | ⚫ |  |  |  |  | ⚫ |  |  |  |
| Care which is tailored around the individual patient |  |  |  |  | ⚫ |  |  | ⚫ |  | ⚫🗶 |  |  |  | 🗶 |  |
| Patients valued that the hospices facilitated activities which were suited to their capabilities which ultimately enabled them to build upon old skills and develop new ones. Carers often watched staff members work so that they could learn new techniques and improve upon their caring capabilities. |  |  | 🗶 |  |  |  | ⚫ |  | 🗶 | ⚫🗶 | ⚫ |  |  | 🗶 |  |
| Carers need validation, to know that they were providing their loved one with the best possible care. | 🗶 |  |  |  |  | 🗶 |  |  |  | 🗶 |  |  |  | 🗶 |  |
| Carers valued household support (laundry etc) as it helps alleviate their burden. |  |  |  |  |  |  |  |  | 🗶 | 🗶 |  |  |  |  |  |

|  | **Key** |
| --- | --- |
| 🗶 | Carer |
| ⚫ | Patient |
