# Supplementary material for: What do patients and family-caregivers value from hospice care? A systematic mixed studies review
Source: BMC Palliat Care. 2019 Feb 8;18:18. doi: 10.1186/s12904-019-0401-1 (PMC6368799; doi:10.1186/s12904-019-0401-1)
Supplement: Supplementary file 6 — CERQUAL, Confidence in qualitative findings table (DOCX 30 kb) [file 12904_2019_401_MOESM6_ESM.docx]

| **Additional file 6:** *CERQUAL Assessment. Summary of qualitative findings* | |  |  |  |  |  |
| --- | --- | --- | --- | --- | --- | --- |
| Summary of review finding  What patients and staff valued | **Studies**  **contributing to**  **the review**  **finding** | **Methodological limitations** | **Coherence** | **Adequacy** | **Relevance** | **CERQual assessment of confidence in the evidence** |
| 1. *Highly skilled staff members*   Knowing that hospice staff had specialised knowledge and experience put patients and informal-caregivers at ease. Patients in particular believed that access to specialist support were more appropriate to anticipate and meet their health needs. | Hyde et al, 2011; Low et al, 2005; Jack et al, 2016; Jack et al, 2014; Borland et al 2014; Holdsworth, 2015;  Hastie et al 2007; Hastie 2009; Jenkins and Codling, 2011; Hastie et al 2005; McLaughlin, 2007. | No or very minor concerns with the methodological limitations of this review finding. 11 studies contributed to this review finding.  Three qualitative studies failed to adequately consider the relationship between researcher and participants (the researcher was often a staff member at the research site). Most of the qualitative studies did not use triangulation or member checking to check the validity of their findings. Whilst there were some limitations in methodology, specifically limitations in the credibility of the primary studies, it was concluded that this did not have a significant effect on this qualitative finding and therefore was categorised as having no or very minor concerns. | No or very minor concerns as it is considered to be well grounded in data from the contributing studies. | Minor concerns. 11 studies contributed to this review finding. 5 studies contributed very little information to this review finding as the skills of staff were only mentioned briefly. Six of the studies provided more detailed information and provided a more exploratory view. Taking into consideration the richness and the quantity of the data (Most single site studies), | No or very minor concerns. Whilst the aims of the included primary studies do not directly answer the research question posed by this review, it was possible to determine important values. In some cases, values could be inferred. All included studies included a high proportion of white British participants, therefore the relevance of this finding to other ethnic groups. | High Confidence |
| 1. *Developing good relationships with health-care professionals*   The development of a close rapport with staff was valuable to both the patient and their family caregiver. These relationships were ameliorated by the health care personnel’s personal traits, experiences and flexible boundaries. | Borland et al, 2014; Hayle et al 2013; Holdsworth 2015; Jack et al 2016; Gambles et al 2002  McKay et al 2013; McLaughlin et al, 2007. | Minor concerns with the methodological limitations of this review finding. Seven studies contributed to this review finding. None of the qualitative studies (n=5) used triangulation or member checking and a lack of research reflexivity was evident in three of the studies. One study reduced the bias associated with their role by excluding all participants known to them. This body of evidence was seen to have minor concerns because the lack of reflexivity and triangulation of methods was not considered to affect this qualitative review finding. Ethical issues were not adequately discussed in two of the studies. Both failed to discuss whether approval had been sought form the ethics committee however, some mention was made to the use of anonymization and measures to minimise distress. Two studies were found to not have an acceptable response rate. | No or very minor concerns as it is considered to be well grounded in data from the contributing studies. | Minor concerns. Seven studies contributed to this review finding. The information from the primary studies were largely descriptive however, one study provided more of an exploratory view. The connection between relationships and staff traits was fairly thin within one study. It was decided that a valid interpretation of the meaning was achieved but due to minor issues with quantity. | No or very minor concerns. Whilst the aims of the included primary studies do not directly answer the research question posed by this review, it was possible to determine important values. In some cases, values could be inferred. All included studies included a high proportion of white British participants, therefore the relevance of this finding to other ethnic groups. | High Confidence |
| 1. *Awareness of patient and carer needs*   Participants referred to the added value of staff members being aware of both the needs of patients’ and their families. | Borland et al, 2014; Jack et al 2016; Exley and Tyrer, 2005 | No or very minor concerns with the methodological limitations of this review finding. Three studies contributed to this review findings. Researcher reflexivity was not considered in two of the studies. One study was found to adequately discuss the ethical issues associated with the study but failed to name the review board where approval was obtained. Whilst there were some concerns with the rigour of analysis in one study due to a lack of information, minor concerns regarding the rigour of data analysis in the other two studies were considered sufficient as multiple researchers were involved in the analysis. It was concluded that there were no or very minor concerns. | No or very minor concerns as it is considered to be well grounded in data from the contributing studies. | Minor concerns. All three studies offered little information. Only a small quantity of studies contributed to this finding. Additionally, it was concluded that there was also a lack of data richness as the importance of awareness was inferred from the data. However due to the simplicity of the finding, it was concluded that there were minor concerns about data adequacy. | No or very minor concerns. Whilst the aims of the included primary studies do not directly answer the research question posed by this review, it was possible to determine important values. In some cases, values could be inferred. All included studies included a high proportion of white British participants, therefore the relevance of this finding to other ethnic groups. | Moderate Confidence |
| 1. *Continuity of care*   Continuity of care refers to both management and relationship continuity. For management continuity, patients and carers valued that the hospice took on a co-ordinating role within and between other healthcare agencies. In reference to relationship continuity, this was facilitated by regular contact by the same professionals. Carers also valued having a smooth transition from pre-post bereavement to avoid abrupt discontinuance of services. | Borland et al, 2014; Hayle et al 2013; Carlebach and Shucksmith, 2010; Holdsworth 2015.  McKay et al 2013; Field et al, 2007; McLaughlin et al 2013; Skilbeck et al, 2005; Lucas et al, 2008 | No or very minor concerns regarding with the methodological limitations of this review finding. Nine studies contributed to this review finding. Two of the studies failed to name the ethics board who approved their study but did sufficiently consider other ethical issues such as informed consent. There were minor concerns regarding researcher bias. Whilst two studies were found to have not adequately considered researcher reflexivity (although one study involved two researchers during the analysis), the other two studies acknowledged the potential bias associated with a dual role as health care professional and researcher. One study attempted to negate this bias by excluding participants known to them. The body of evidence supporting the review finding was assessed as having no or very minor concerns. | No or very minor concerns as it is considered to be well grounded in data from the contributing studies. | Moderate concerns. The contributing studies all had relatively small sample sizes and had relatively thin data which gives cause for concern. However, due to the descriptive and simplistic nature of the finding, it was concluded that there were moderate concerns regarding adequacy. | No or very minor concerns. Whilst the aims of the included primary studies do not directly answer the research question posed by this review, it was possible to determine important values. In some cases, values could be inferred. All included studies included a high proportion of white British participants, therefore the relevance of this finding to other ethnic groups. | Moderate Confidence |
| 1. *Social opportunities helped to develop relationships with other patients and carers*   The hospice provided formal opportunities for patients and carers to develop relationships with others and create a support network independent of the family. | Hyde et al 2011; Low et al 2005; Woolf and Fisher, 2015; Hayle et al 2013; Williams and Gardener; Kennett et al, 2005; Kennett, 2000; Hopkinson and Hallett, 2001  Goodwin et al 2002; Kernohan et al 2006; Hastie et al 2007; Hastie et al 2009. | Minor concerns with the methodological limitations of this review finding. 12 included articles contributed to this review finding. Limitations in researcher reflexology evident in five of the studies with one failing to mention bias associated with a dual role (Researcher and staff member at the research site). Four studies were judged to have not adequately discussed the ethical implication of their study. One study made no mention of anonymisation of transcripts or how consent was taken. None of the studies used triangulation or member checking to check the validity of their findings and three studies were found to have not given enough detail regarding their analysis process. Whilst limitations were evident, it was concluded that there were minor concerns regarding methodological limitations. | Minor concerns. Although the data from the contributing studies was largely consistent across all the studies, discrepancies occurred when in relation to social support for carers. Carers often noted that they would take advantage of informal social support opportunities and no mention was made to official social opportunities. | Minor concerns. This finding was based on 12 studies. Whilst some studies offered little information regarding this phenomenon, the richness of the data from the other contributing studies along with the descriptive nature of the finding, it was concluded that there were no or very minor concerns. | No or very minor concerns. Whilst the aims of the included primary studies do not directly answer the research question posed by this review, it was possible to determine important values. In some cases, values could be inferred. All included studies included a high proportion of white British participants, therefore the relevance of this finding to other ethnic groups. | High Confidence |
| 1. *Maintaining a sense of normality*   *The provision of support to enable a sense of normality to remain for both patients and carers was a recurrent theme. This sense was often encouraged through maintaining and creating relationships within and outside of the hospice and the provision of respite. Patients often valued the opportunity to escape their sick role which was supported by being made to feel like an autonomous individual and remain connected to their self-identity.* | Hyde et al 2011; Jack et al 2014; Jack et al 2016; Thomas 2001; Hopkinson and Hallett, 2001  Goodwin et al 2002; | Minor concerns with the methodological limitations of this review finding. Six studies contributed to this review finding. Two studies did not adequately consider the relationship between researcher and participants. The recruitment strategy in one study was found to be inappropriate, not only due to a lack of detail surrounding the recruitment process but also due to the recruitment of a small sample of participants. A lack of triangulation and member checking in addition to a lack of detail surrounding the analysis of data resulted in the conclusion that there were minor concerns regarding the methodological limitations associated with this qualitative review finding. | No or very minor concerns as it is considered to be well grounded in data from the contributing studies. | Moderate concerns. Whist the quantity of the data was deemed sufficient, the richness of the data was absent. The data that this finding was based on offered little information about this phenomenon, therefore it was not possible to explore the importance of normality to hospice service users. However, due to the descriptive nature of this finding, it was concluded that there were moderate concerns. | No or very minor concerns. Whilst the aims of the included primary studies do not directly answer the research question posed by this review, it was possible to determine important values. In some cases, values could be inferred. All included studies included a high proportion of white British participants, therefore the relevance of this finding to other ethnic groups. | Moderate Confidence |
| 1. *Help to develop old and new skills*   Patients sometimes felt that they had lost the ability to continue with previous hobbies. This resulted in the reduction of feelings of self-worth and independence. Patients valued having the opportunity to continue on with their hobbies which had been altered to better suit their abilities. In some instances, patients were able to learn new skills/hobbies which helped them to regain a sense of normality. | Kennett 2000; Woolf and Fisher, 2015;  Goodwin et al, 2002 | Moderate concerns with the methodological limitations of this review finding. Three studies contributed to this review finding. Triangulation or member checking not evident in both studies. One study lacked detail when discussing the ethical implications of the study. Whilst it was stated that they had approvals from their local ethics committee (not named), other ethical considerations such as informed consent, anonymization, and support were not referenced. Due to the limited number of studies contributing to this finding, in addition to issues in the recruitment strategies, small sample of participants and the lack of detail surrounding the ethical considerations in one study It was concluded that there were moderate concerns regarding this review finding. | No or very minor concerns as it is considered to be well grounded in data from the contributing studies. | Serious concerns. Whilst this finding is largely descriptive, there were serious concerns with both the quantity and richness of the data. Due to these issues we could not conclude that we had a good understanding of this phenomenon. | No or very minor concerns. Whilst the aims of the included primary studies do not directly answer the research question posed by this review, it was possible to determine important values. In some cases, values could be inferred. All included studies included a high proportion of white British participants, therefore the relevance of this finding to other ethnic groups. | Moderate Confidence |
| *Accessibility and availability of the hospice and hospice services* | | | | |  |  |
| 1. *Access to a wide range of services and staff*   The accessibility and availability of the hospice and everything it encompasses was especially important to both patients and their family caregivers. Particular focus was placed on access to out-of-hours support, the ability to participate in a wide range of activities suited to their needs and abilities and open visiting hours. | Jack et al 2014; Low et al 2005; Kennett 2000; Woolf and Fisher, 2015; Thomas 2001; Exley and Tyrer, 2005; Carlebach and Shucksmith, 2010; Holdsworth, 2015  Hopkinson and Hallett, 2001; Gambles et al, 2002;  McKay et al 2013; Goodwin et al 2002; Kernohan et al, 2007; Hastie et al, 2007; Hastie et al, 2009; Jenkins and Codling, 2011; Hastie et al, 2005. | Minor concerns with the methodological limitations of this review finding. Seventeen studies contributed to this review finding. Eight studies did not adequately reflect upon researcher reflexivity and five studies did not adequately reflect upon the ethical implications of their study. The body of evidence supporting this review finding was assessed as having no or very minor concerns. | Moderate concerns regarding the consistency of this review finding. Inconsistencies in the data were evident in relation to open visiting hours and access to out of hours support. Whilst some participants valued open visiting hours, for some it resulted in a loss of autonomy. The value of out-of-hours support for patients and families utilising the hospice at home service was describable. Some participants in the contributing studies felt that they did not have adequate access to out–of-hours support. | Minor concerns. Whilst seventeen studies contributed to this qualitative finding, only six of the studies were considered to provide rich and detailed information specific to the phenomenon of interest. These studies helped us to understand and explore the importance of availability and accessibility. The remaining four contributing articles often discussed the benefits of a particular service but did not directly refer to the importance of availability of the particular service resulting in the need to infer this value from the data. Based on the overall assessment of the richness and quantity of the data, it was concluded that there were minor concerns with the adequacy of this review finding. | No or very minor concerns. Whilst the aims of the included primary studies do not directly answer the research question posed by this review, it was possible to determine important values. In some cases, values could be inferred. All included studies included a high proportion of white British participants, therefore the relevance of this finding to other ethnic groups. | High Confidence |
| 1. *Time*   Staff at the hospice were able to significantly spend more time with patients. This enabled opportunities for patients to ask question about their care and have sufficient answers. The time spent with other patients was also considered invaluable. | Hopkinson and Hallett, 2001. | Moderate concerns with the methodological limitations of this review finding. One study contributed to this review finding. Issues with researcher reflexivity, ethical considerations and the rigour of the data analysis was evident within this study. As a result, this qualitative finding was found to have moderate methodological limitations. | No or very minor concerns as it is considered to be well grounded in data from the contributing studies. | Serious concerns. Whilst this finding is largely descriptive, there were serious concerns with both the quantity and richness of the data. Due to these issues we could not conclude that we had a good understanding of this phenomenon. | No or very minor concerns. Whilst the aims of the included primary studies do not directly answer the research question posed by this review, it was possible to determine important values. In some cases, values could be inferred. All included studies included a high proportion of white British participants, therefore the relevance of this finding to other ethnic groups. | Moderate Confidence |
| 1. *Hospice atmosphere*   The friendly and welcoming atmosphere of the hospice facility ensured that all patients and their families/caregivers felt safe, relaxed and welcome. For those receiving care at home, the welcoming environment was engendered by the hospice staff. | Low et al 2005; Hayle et al 2013; Exley and Tyrer, 2005); Hopkinson and Hallett, 2001; Gambles et al, 2002;  Hastie et al 2007; Hastie et al 2009; Jenkins and Codling 2011; Hastie et al, 2005. | Minor concerns with the methodological limitations of this review finding. Nine studies contributed to this finding. Researcher reflexivity was not adequately considered in four studies. None of the studies did not rigorously report on their data analysis. Finally, three studies did not report enough detail regarding the recruitment process in their study. The body of evidence supporting this review finding was assessed as having minor concerns. | No or very minor concerns as it is considered to be well grounded in data from the contributing studies. | Serious concerns. Of the nine contributing studies, eight did not provide enough detail to get a rich understanding of the phenomenon. Whilst the final study did provide more detail, due to issues with the richness of the information in the latter, along with issues in quantity, it was concluded that we had serious concerns regarding the adequacy of this finding. Whilst the finding is largely simple and descriptive it was unclear how a welcoming environment was facilitated, who contributed positively to this environment, why it was of value or how important it was. | No or very minor concerns. Whilst the aims of the included primary studies do not directly answer the research question posed by this review, it was possible to determine important values. In some cases, values could be inferred. All included studies included a high proportion of white British participants, therefore the relevance of this finding to other ethnic groups. | Moderate Confidence |
| 1. *Maintenance of psychological, spiritual and physical well- being*   The right balance of physical, psychological and spiritual support enhanced the quality of life for both patients and carers. | Jack et al 2016; Kennett 2000; Woolf and Fisher 2015; Thomas, 2001; Hayle et al 2013; Holdsworth, 2015;  Hopkinson and Hallet, 2001; Gambles et al, 2002);  McLaughlin et al, 2013. | Minor concerns with the methodological limitations of this review finding. Nine studies contributed to this finding. Of these nine, four studies did not adequately consider the relationship between researcher and participants.  Two studies did not rigorously analyse their data and three studies failed to adequately discuss the ethical implication of their study. One study was found to not have an acceptable response rate. | This review finding was assessed as having no or very minor concerns as it is well grounded in data from the contributing studies. | Moderate concerns. Most of the studies contributing to this review finding referred to the importance of symptom control as an outcome of a service but was not directly mentioned as a value. For example, one study explained how a lack of control when it came to visit resulted in a negative effect on physical symptoms. From this, it was inferred that physical well-being was important. One study provided a rich explanation as to the phenomenon. Considering the quantity and the richness of the data, we concluded that there were moderate concerns. | No or very minor concerns. Whilst the aims of the included primary studies do not directly answer the research question posed by this review, it was possible to determine important values. In some cases, values could be inferred. All included studies included a high proportion of white British participants, therefore the relevance of this finding to other ethnic groups. | High Confidence |
| 1. *Promoting patient and carer independence through choice*   The hospice helped to facilitate independence by helping to promote choice. The promotion of choice was facilitated in numerous ways. This included support to ensure patients die in their preferred place, enabling patient to decide whether they want to partake in activities and involving them in the decision- making process. | Hyde et al 2011; Jack et al 2016; Low et al 2005; Kennett 2000;  Thomas et al; Holdsworth, 2015; Hopkinson and Hallett, 2001;  McKay et al 2013; McLaughlin et al, 2007; Hastie et al, 2007; Hastie et al 2009; Jenkins and Codling 2011; Hastie et al, 2005. | No or very minor with the methodological limitations of this review finding. 13 studies contributed to this finding. Limitations were evident in researcher reflexivity in two studies. Five of the studies did not include enough detail on the rigour of the data analysis process. The ethical implications of the studies were not adequately described in two studies. Limitations in the recruitment process was evident in five studies. The body of evidence supporting the review finding was found to have no or very minor concerns. | Minor concerns as it is considered to be well grounded in data from the contributing studies. Although some studies referred to a lack of patient autonomy due to open visiting hours. | Minor concerns. These studies described how having choice was important. Whilst the data from the contributing studies was relatively thin, due to the quantity and the descriptive and simplistic nature of the finding, it was concluded that there were only minor concerns with the adequacy of this review finding. | No or very minor concerns. Whilst the aims of the included primary studies do not directly answer the research question posed by this review, it was possible to determine important values. In some cases, values could be inferred. All included studies included a high proportion of white British participants, therefore the relevance of this finding to other ethnic groups. | High Confidence |
| 1. *Practical support for patient and carers*   Practical support meant ensuring patients and carers were signposted to the correct agencies, that they had access to equipment, domestic support and help when patients needed moving. | Borland et al 2014; Jack et al 2016; Exley and Tyrer, 2005;  McKay et al, 2013; McLaughlin et al 2013; Lucas et al, 2008. | No or minimal concerns with the methodological limitations of this review finding. Six studies contributed to this review finding. One study did not adequately reflect upon the relationship between participants and relationships. One study was also found to have limitations in the description of the analysis process. This finding was found to have no or minimal methodological limitations. | No or very minor concerns as it is considered to be well grounded in data from the contributing studies. | Moderate concerns. Whilst the data within five of the contributing studies was thin, due to the richness of the final paper along with the descriptive nature of the finding, it was concluded that there were moderate concerns with the adequacy of this review finding. | No or very minor concerns. Whilst the aims of the included primary studies do not directly answer the research question posed by this review, it was possible to determine important values. In some cases, values could be inferred. All included studies included a high proportion of white British participants, therefore the relevance of this finding to other ethnic groups. | Moderate Confidence |
| 1. *Preparation*   Open discussions with staff ensured that both patients and carers were prepared for death which reduced the fear of the unknown. Information about their illness, and predicted illness trajectory further facilitated a sense of preparedness. | Borland et al 2014; Jack et al, 2014; Low et al 2005; Hayle et al 2013; Exley and Tyrer 2005; Holdsworth, 2015 | Minor concerns with the methodological limitations of this review finding. Six studies contributed to this review finding. Reflexivity was an issue in three of the included studies. The analysis of the data was found to not be sufficiently rigorous. Researchers failed to provide enough detail regarding their analysis process in all but one study. Sufficient ethical consideration was absent from one study as they failed to provide any information regarding any ethical considerations. This finding was found to have minor methodological limitations. | No or very minor concerns as it is considered to be well grounded in data from the contributing studies. | Minor concerns. Only two of the included studies provided a rich exploration of the phenomenon of interest. However, due to the descriptive nature of the review finding along with the richness of the data within the two studies, it was concluded that there were minor concerns with the adequacy of this review finding. | No or very minor concerns. Whilst the aims of the included primary studies do not directly answer the research question posed by this review, it was possible to determine important values. In some cases, values could be inferred. All included studies included a high proportion of white British participants, therefore the relevance of this finding to other ethnic groups. | High Confidence |
