# Supplementary material for: What do patients and family-caregivers value from hospice care? A systematic mixed studies review
Source: BMC Palliat Care. 2019 Feb 8;18:18. doi: 10.1186/s12904-019-0401-1 (PMC6368799; doi:10.1186/s12904-019-0401-1)
Supplement: Supplementary file 7 — A list of the seven articles that could not be accessed (DOCX 16 kb) [file 12904_2019_401_MOESM7_ESM.docx]

**Additional file 7*:*** *A list of articles that could not be accessed*

1. Beach, M.Patient experience key in hospice refurb. Health Estate. 2015; 69:47-51.
2. Blaney, P, Mulholland C, Molloy, A. Let’s talk about-all Ireland survey of palliative care experiences. BMJ Support Palliative care. 2015; 5.
3. Boyd, K. Short terminal admissions to a hospice. Palliative Medicine. 1993; 7: 289-294.
4. Cheesman, S, Christian R, Cresswell J. Exploring the value of shiatsu in palliative care day services. International Journal of Palliative nursing. 2001; 7: 234-9.
5. Corr CA, Corr DM. Adult hospice day care. Death Studies.1992; 16:155-71.
6. Donnelly SM, Donnelly CN. The experience of the moment of death in a specialist palliative care unit (SPCU). Irish Medical Journal. 2009; 102: 143-6.

1. Emanuel L, Alpert HR, Baldwin DC, Emanuel EJ. What terminally ill patients care about: toward a validated construct of patients’ perspectives. Journal of Palliative care. 2000; 3:419-31.
