# Supplementary material for: What do patients and family-caregivers value from hospice care? A systematic mixed studies review
Source: BMC Palliat Care. 2019 Feb 8;18:18. doi: 10.1186/s12904-019-0401-1 (PMC6368799; doi:10.1186/s12904-019-0401-1)
Supplement: Supplementary file 8 — Quotations from included studies and their corresponding theme. (DOCX 15 kb) [file 12904_2019_401_MOESM8_ESM.docx]

| **Additional file 8:** A *sample of quotations from included studies* | | | |
| --- | --- | --- | --- |
| **Analytical theme; Descriptive theme** | | **Quote number** | **Quote** |
| The importance of staff in the provision of high standard quality care | **1**  **2**  **3** | | *“It did help, ‘cause I knew she was in good hands” (Jack et al 2014, 135).*  *“I mean you can’t believe that a doctor would give that amount of time” (Holdsworth 2015, 837).*  *``nothing's too much trouble for them'' and ``I don't think anyone could go out of their way more than they do'' (Hopkinson and Hallett 2001, 120).* |
| The role of social engagement and participation in social activities in the maintenance of relationships and their sense of normality; Peer support provided a support network | **4** | | *“If I was trying to explain to my wife how I was feeling she’d need therapy. Here people understand” (Low et al 2005, 67).* |
|  | **5**  **6** | | “*he had had no emotional support during his treatment and said that all of a sudden, he could not do a thing but that coming to the hospice had brought about a change”*  *“help other people despite your symptoms. Reassure them you know how they feel” (Hopkinson and Hallett 2001, 121).* |
| The comfort gained from the availability and accessibility of the hospice; availability of the hospice | **7**  **8**  **9** | | “*instead of having it all regimental like”* (Hopkinson and Hallett 2001, 121).  *“If I didn’t have that service… I would probably feel very, well more vulnerable than what I felt”*  *“I do think it was good that they were there and I could ring them. I always had their number and I did ring them. I found it reassuring that I had a source that I could ring at any given moment” (Borland et al 2014, 707).* |
|  | **10** | | *It takes the weight off …. Someone is there to help you… I don’t feel frightened if [name of patient] isn’t very well now. I know I can phone someone and they will give me some advice” (Low et al 2005, 68).* |
|  | **11** | | *“I valued that somebody was here the night he died, because the nurse called us when she thought it was time, so my daughter and I were with him”* (Jack et al 2016, 135). |
|  | **12** | | *“We might chat or just sit and hold hands… We’ve both been asleep in the ward. I was asleep in the bed and she was sat next to me in the chair asleep and we were holding hands”* (Thomas 2001, 501). |
|  | **13** | | *“Nurses knowing, I needed artificial saliva for night time use. It was very practical, and I’d never heard of it before and it’s invaluable” (Jack et al 2016, 2167)*. |
| The role of the hospice in helping promote patient and carer autonomy through the provision of various support mechanisms | **14** | | *“I want to die at home. I desperately want to die at home but without this [Hospice at Home Service] I’d have probably ended up in some nursing home somewhere” (Jack et al 2016, 2168).* |
|  | **15** | | *“if you want to do something well they start helping you, if you say you want to do it. . .Or if you don't, `ahh, I'll have forty winks', well they leave you and you have forty winks, you know. And it's nice to be able to do that” (Hopkinson and Hallett 2001, 121).* |
